# Lipopeptides for Vaccine Development

**DOI:** 10.1021/acs.bioconjchem.1c00258

**Published:** 2021-07-06

**Authors:** Ian W. Hamley

**Affiliations:** Department of Chemistry, University of Reading, Whiteknights, Reading RG6 6AD, U.K.

## Abstract

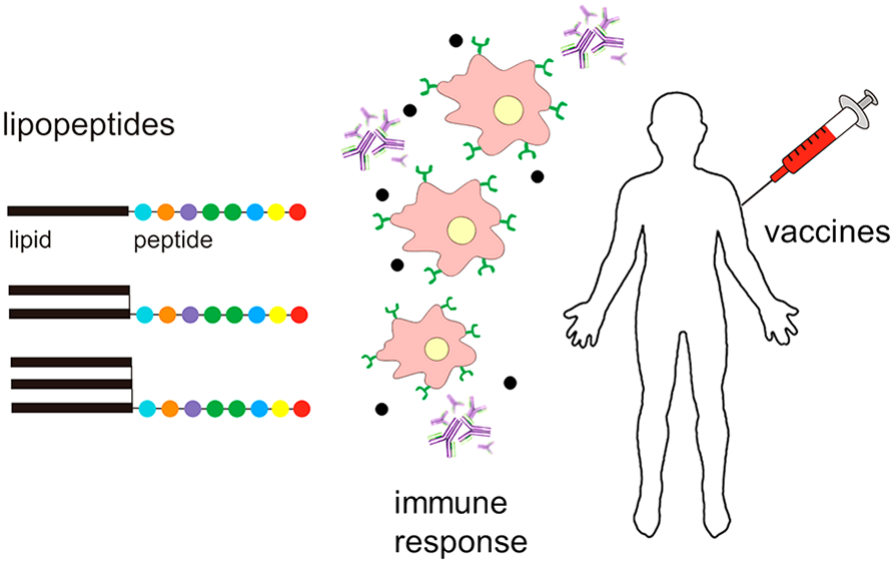

The development of lipopeptides (lipidated peptides) for vaccines is discussed, including their role as antigens and/or adjuvants. Distinct classes of lipopeptide architectures are covered including simple linear and ligated constructs and lipid core peptides. The design, synthesis, and immunological responses of the important class of glycerol-based Toll-like receptor agonist lipopeptides such as Pam_3_CSK_4_, which contains three palmitoyl chains and a CSK_4_ hexapeptide sequence, and many derivatives of this model immunogenic compound are also reviewed. Self-assembled lipopeptide structures including spherical and worm-like micelles that have been shown to act as vaccine agents are also described. The work discussed includes examples of lipopeptides developed with model antigens, as well as for immunotherapies to treat many infectious diseases including malaria, influenza, hepatitis, COVID-19, and many others, as well as cancer immunotherapies. Some of these have proceeded to clinical development. The research discussed highlights the huge potential of, and diversity of roles for, lipopeptides in contemporary and future vaccine development.

## 1 Introduction

Vaccination has been a highly successful life-saving method to prevent viral infections, since its development by Jenner in 1796 to treat smallpox, which has now been eradicated. Vaccines based on live, attenuated pathogens have been developed for many diseases; however, for other infections, such vaccines have not been successful or they are ineffective since they fail to provide long-lasting immunity (for example, influenza). In this case, vaccines that use inactivated viruses or other subunits may be created. The current COVID-19 pandemic has focused attention on the impact of viral disease on human society and already vaccines have been developed, remarkably rapidly, based on mRNA technology (the genetic material is delivered in liposomes) or the use of genetically modified (nonhuman) adenovirus carriers (which incorporate spike protein genes), or simply nanoparticle formulations containing virus spike protein subunits as well as whole inactivated viruses. Further information on this is widely available.^1−6^ Peptides and lipopeptides are attractive in the development of vaccines, both as antigens and as adjuvants. Peptide and lipopeptide antigens can be developed based on sequences from antigenic proteins, and they can be used to stimulate cell surface receptors in a highly specific manner. Peptides offer advantages in the ease of design and preparation using automated synthesis methods, and they can also be conjugated to lipids and other molecules in the development of adjuvants (or for antigen presentation) in subunit vaccines. Conjugates of peptides and lipids, giving lipidated peptides, termed lipopeptides, are the focus of the current Review which discusses various approaches to the synthesis and application of these molecules, a type of peptide amphiphile. Peptides are relatively inexpensive and safe to produce and can be synthesized at high purity, avoiding contaminants such as lipopolysaccharides that can be present in bioderived protein materials. A further feature of certain classes of peptide-based molecules including lipopeptides is their propensity to self-assemble into nanostructures such as fibrils, micelles, and other structures.^7−13^ This property is of interest because it leads to presentation of bioactive peptide motifs at high density, leading potentially to improved antigen and/or adjuvant efficacy, and some examples utilizing this strategy are discussed in this Review. As yet, lipopeptide-based vaccines are not used in clinical practice, although several peptide-based vaccines have reached clinical trials and/or are currently in active development.^14−19^ Many vaccines require formulation with adjuvants, which are biomolecules that stimulate immune responses in order to enhance the activity of a vaccine. The roles of lipopeptides as antigens and/or adjuvants is the focus of the current Review, and it includes the important class of self-adjuvanting lipopeptides, which incorporate both antigen and adjuvant activities. This Review does not consider proteins or lipoproteins and is focused on lipopeptides. Although there is no rigorous distinction between long peptides and proteins, here we consider peptide-based systems with less than about 100 residues.

Potential treatments for cancer also include novel immunotherapies. Cancer immunotherapies include potential vaccines, monoclonal antibodies, T cell transfer therapy including CAR (chimeric antigen receptor) T cell therapy, immune checkpoint inhibitors (which modulate immune response), and immune system modulators such as cytokines (for example, interferons and interleukins). Lipopeptides have potential roles in many of these approaches; in particular, TLR (Toll-like receptor) agonist peptide-based molecules have attracted attention in cancer immunotherapies, as discussed in more detail here, and in a recent review focused on lipopeptides with applications as adjuvants for cancer vaccines.^20^

This Review is organized as follows. Section 2 provides a brief overview of the immune system and provides the background on the cellular processes associated with the immune response, as well as introducing key terminology. Section 3 concerns ligated lipopeptides, in which lipid chains and peptides are coupled by ligation methods. Section 4 covers TLR agonist lipopeptides, one of the most intensely researched types of peptide amphiphiles, because some of this class provoke a strong and specific immune response, and indeed such molecules are used as model TLR agonists. Section 5 discusses lipid core peptides which have more complex architectures than those of ligated and TLR agonist lipopeptides, indeed generally offering a multivalent presentation of peptide epitopes. Lipopeptide micelle self-assembled structures relevant to immunotherapies are discussed in section 6. Concluding remarks in section 7 close this Review.

## 2 Brief Overview of the Immune System, Introducing Key Terms

**Figure 1:**
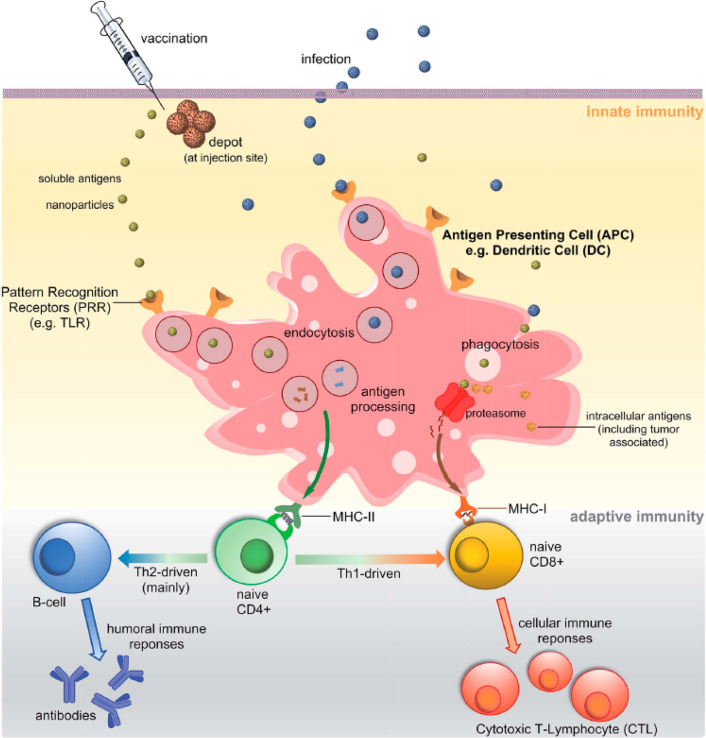
Cell types and other structures and processes involved in innate and adaptive immunity, which are referred to in this Review. From ref (18). Published by the Royal Society of Chemistry.

The immune system comprises the innate and adaptive systems. The innate immune system relies on macrophages, neutrophils, natural killer cells, dendritic cells, and others. The adaptive immune system is activated by the presentation of antigens by antigen-presenting cells (APCs) of the innate immune system. Antigen presentation involves the binding of antigen to the major histocompatibility complex (MHC), the complex then being transported to the cell surface where it is presented and is capable of recognition by a T cell receptor (TCR). This receptor binds peptides presented by class I or class II major histocompatibility complexes (also known as human leukocyte antigen, HLA, for humans) on APCs (Figure 1). Whole antigens are processed by proteolysis by APCs into short peptides (8–11 residues in length for class I MHCs and 11–30 residues in length for class II MHCs), which are presented via MHCs at APC surfaces.^19^ TCRs that are specific for particular peptide epitopes then bind the peptide–MHC complexes, and a variety of proteins at the T cell/APC interface orchestrate expansion of clones of the T cells.

There are two types of MHC, one is involved with intracellular peptides from the cytosol (MHC class I), while MHC class II molecules bind peptides in endocytotic vesicles after internalization. The MHC-I/peptide complex activates cytotoxic T cells (T_c_ or killer T cells or cytotoxic T-lymphocyte CTL, a type of white blood cell) via TCRs, these cells also expressing CD8^+^ co-receptors (Figure 1) (here, CD refers to cluster of differentiation, a class of cell surface glycoproteins that can act as receptors or ligands). On the other hand, presentation of antigens via MHC class II is to CD4^+^ T cells (T_h_ helper T cells, another type of white blood cell which sends signals to other cells including T_c_ cells) (Figure 1). Only specific types of APCs such as dendritic cells, B cells, or macrophages present MHC-II at high levels, so expression of MHC-II molecules is more cell-specific than MHC-I.

The innate immune system relies on pattern recognition receptors (PRRs) to detect infecting microbes (Figure 1). PRRs are present in germline cells. They can detect a wide range of pathogens, although they lack the specificity of somatic T and B cells. Most adjuvants are ligands for PRRs. The innate immune response involves the recognition by PRRs of pathogen-associated molecular patterns (PAMPs) which include TLRs, NOD-like receptors (NLRs), C-type lectin agonists (CLRs), RIG-I (retinoic acid-inducible gene I), stimulator of interferon (IFN) genes (STINGs), and others.^22−24^ Adjuvants based on these and other PAMPs can enhance the immune response (act as agonists) by interacting with PRRs on antigen-presenting cells. PAMPs elicit specific antigen presentation and cytokine production. The role of TLRs which are a key target for lipopeptides, as discussed later in this Review, has been reviewed extensively.^25−28^ PRRs signal through a variety of pathways involving distinct intermediates and key transcriptional factors NF-κB (nuclear factor kappa-light-chain-enhancer of activated B cells), and interferon regulatory factors IRF-3 and IRF-7 (IRFs primarily regulate type I interferons, IFNs, in the host after pathogen infection) are activated, leading to the production of cytokines and chemokines that prime and expand the immune response.

The activated adaptive immune system relies on antigen-recognizing species including T cells, B cells, dendritic cells, and antibodies (immunoglobulins). As mentioned above, the adaptive immune system produces T-helper cells which release cytokines to “help” other immune cells. T-Helper cells proliferate into differentiated Th1 cells or Th2 cells (Figure 1). The former lead to a cell-mediated response and the latter to a humoral response which refers to the production of antibodies and antimicrobial peptides and other agents in extracellular fluid (and is also known as antibody-mediated immunity). The main effector cells of Th1 immunity are macrophages as well as CD8^+^ T cells, IgG B cells, and IFN-γ CD4^+^ T cells. The main effector cells for Th2 are leucocytes (white blood cells) including basophils, eosinophils, and mast cells as well as B cells and IL-4/IL-5 CD4^+^ T cells. Th1 cells produce cytokines including INF-γ (interferon-gamma) and TNF-β (tumor necrosis factor-beta). The Th2 response leads to interleukin cytokines including IL-4, IL-5, IL-6, IL-9, IL-10, and IL-13. Th1-stimulated IFN-γ increases the production of IL-12 by dendritic cells and macrophages.

The activity of a vaccine may be enhanced using adjuvants, and adjuvant development is currently attracting immense interest as a means to substantially boost the performance of vaccines active against a number of diseases. Many vaccines confer humoral immunity, although adjuvants have been/are being developed to stimulate cellular (Th1) immunity.^22^ The most effective licensed vaccines elicit persistent T cell and B cell memory as well as long-term antigen-specific antibody responses by plasma cells.^29^ Adjuvants are used to increase antibody production or to modulate the adaptive response. They boost the performance of vaccines, enabling a lower dose of antigen and/or a smaller number of required vaccinations. They hence increase the level of immunization within the general population that has been vaccinated and can boost immunity for groups with lowered responsiveness such as older or infant populations.^22^ As well as generally boosting the immune response, adjuvants can be used to adjust the nature of the immune response, for example, to change the Th1 versus Th2 response (so-called polarization of helper T cells) or the balance of cytotoxic T cells versus helper CD4^+^ T cells or T cell memory as well as altering the rate of immune response and its specificity.^22^ The type of adjuvant selected will depend on the nature of the desired CD4^+^ T cell response. Adjuvants should elicit a protective CD8^+^ response depending on the type of vaccine. Vaccines that cause direct infection of cells including viral carriers or RNA/DNA induce CD8^+^ immunity through the endogenous class I presentation pathway; however, other vaccines require cross-presentation.^22^ Adjuvants have a number of roles including enhanced presentation of antigens and direct stimulation of an immune response using inactivated toxin or virus components or other immune-stimulating molecules such as bacterial lipopolysaccharides or Toll-like receptor agonists including lipopeptides, as discussed in section 4.

Traditionally, alum has been used as a common vaccine adjuvant, and recent reviews discuss other widely used inorganic adjuvants; see, for example, refs (22, 23, and 30). Later developments include oil-in-water emulsions such as Freund’s complete adjuvant which contains heat-killed *Mycobacterium tuberculosis* (incomplete Freund’s adjuvant lacks the mycobacteria) or squalene oil-in-water emulsions AS03 or MF59 (squalene is more readily metabolized than paraffin, used in Freund’s adjuvants).^22,23^ Alum and emulsion adjuvants are generally considered to have good safety profiles.^22,31,32^ However, Freund’s aduvant contains many innate immune stimulants and can cause side serious effects; indeed, use of both Freund’s complete and incomplete adjuvants was discontinued for this reason.^23,32−34^

Organic adjuvants such as those based on peptides, proteins, lipids, polysaccharides, and lipopeptides offer scope to tailor more specific immune responses and are the focus of considerable interest in the development of new vaccines. Research in the field of organic vaccine adjuvants has been reviewed.^22,32,35^

## 3 Ligated Lipopeptides

**Scheme 1:**
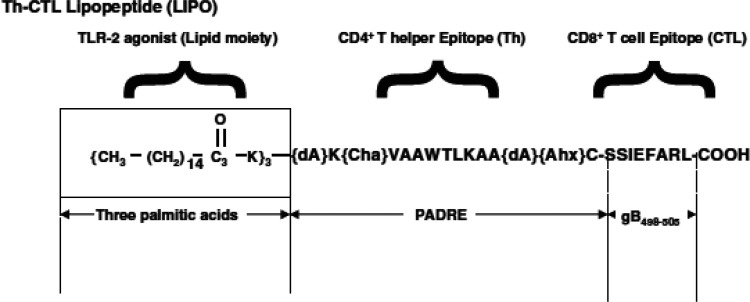
Tri-Palmitoylated Lipopeptides Prepared with Dual-Function Epitopes^36^ The Pan DR peptide (PADRE) is a universal CD4^+^ epitope, and gB_498–505_ indicates the HSV glycoprotein B (gB) CD8^+^ cytotoxic T cell immunodominant epitope. Abbreviations: dA, l-alanine; Cha, l-cyclohexyl alanine; Ahx, aminocaproic acid. Reprinted by permission from Nature Publishing Group.^36^ Copyright 2009.

A series of lipopeptides (sequences given in ref (37)) containing palmitoyl chains at lysine ε-amino groups has been produced as part of an HIV vaccine development program.^37−39^ Although the conformation and self-assembly of these lipopeptides has not been examined, they show promise as vaccines, since B and T cell anti-HIV responses were detected in >85% of the vaccinated volunteers after one month, and the research proceeded to clinical trials.^14,39,40^ In another study, palmitoylation at the lysine residue was also used to increase the immunogenicity of a series of peptides derived from four malaria parasite *P. falciparum* antigens.^41^ Again, although information on peptide and lipopeptide conformation and ordering is not provided, these molecules generate multi-epitopic and long-lasting antigen-specific CD8^+^ CTL responses in chimpanzees, showing potential also as human vaccines.^41^ N-Terminal lipidation (palmitoylation) of the model antigen herpes simplex virus type 1 (HSV-1) gD_1–23_ peptide increases uptake and maturation of dendritic cells via TLR2 and triggers Th1-dependent protective immunity.^42^ The lipidation was performed by N-terminal attachment of a single *N*^ε^-palmitoyl-lysine to the N-terminal lysine ε-amino in the peptide using chemoselective ligation.^42^ This method avoids problems with solid phase lipidation methods that arise due to the amphiphilicity of the lipopeptides which can complicate solution separation of the target lipopeptide.^43^ The method can be used to prepare mixtures of lipopeptides (demonstrated with peptides based on simian immunodeficiency virus fragments and a *Clostridium tetani* sequence) and involves the site-specific introduction of a fatty acyl moiety in solution to a mixture of individually prepurified peptides.^43^ The lipidation is based on the quasi-stoichiometric and high-yield ligation of a glyoxylyl lipid with hydrazinoacetyl peptides. This method was used to produce the HIV lipopeptide cocktails mentioned above, along with other antigen sequences.^44−46^ A modification of this method was used to attach three N-terminal palmitoyl chains (via three K residues) to two peptide epitopes in the development of a herpes simplex virus vaccine for intravaginal delivery via the genital mucosa.^36^ The peptide sequence includes CD4^+^ Th and CD8^+^ CTL sequences (Scheme 1), and the attachment of one, two, or three palmitoyl chains was motivated by the demonstrated TLR2 agonist properties of related Pam_*n*_Cys lipopeptides (discussed in section 4).^36,47^

## 4 Toll-Like Receptor Agonist Lipopeptides

Lipoproteins and lipopeptides are important components of the cell wall of both Gram-negative and Gram-positive bacteria. Gram-negative bacterial membranes tend to contain lipopeptides with three lipid chains, whereas Gram-positive cell walls contain lipopeptides bearing two lipid chains.^28^ These are heterogeneous in terms of fatty acid chain length and degree of saturation. Lipoproteins derived from Gram-negative bacteria, such as *E. coli*,^50^*Borrelia burgdorferi*,^51^*Neisseria gonorrheae*,^52^*Neisseria meningitidis* (*meningococcus*),^53^ and *Porphyromonas gingivalis*,^54^ have been shown to be TLR2 agonist triacylated lipoproteins (*N. meningitidis* lipoproteins are the basis of the vaccine Trumenba^54^). Lipoproteins from Gram-positive bacteria such as *Staphylococcus aureus* act on TLR2 and are diacylated derivatives.^55,56^

**Scheme 2:**
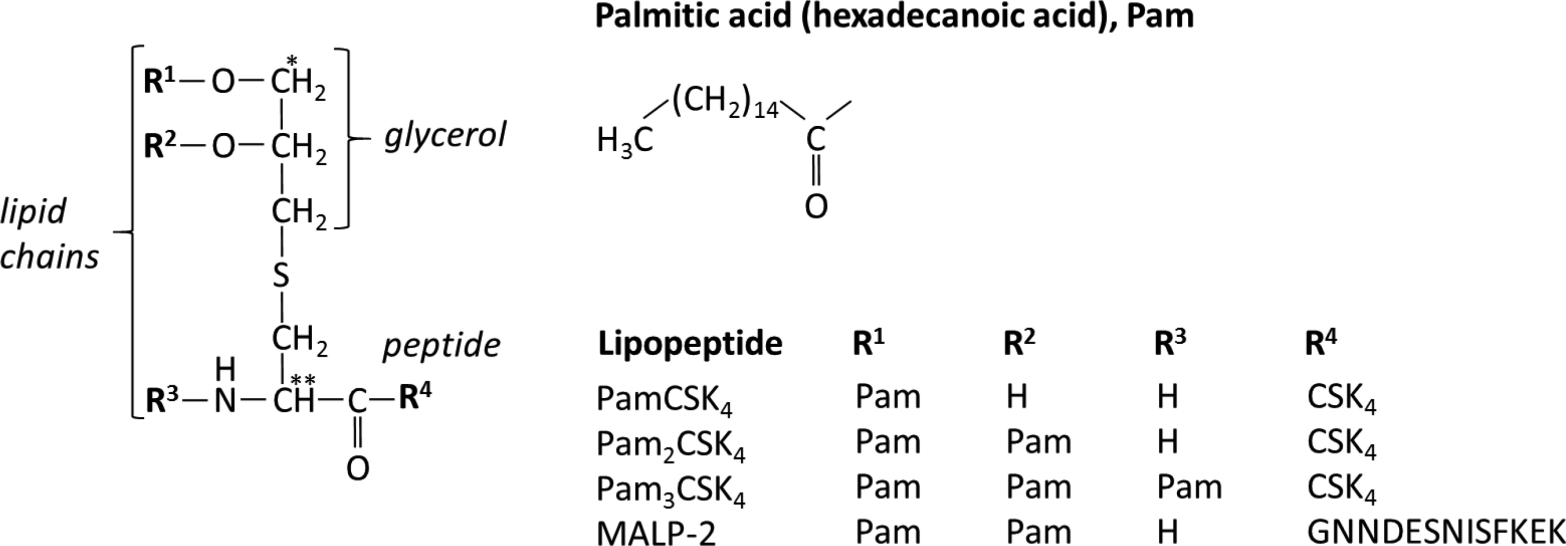
General Schematic of the Structure of Pam_*n*_Cys and MALP-2 Lipopeptides in Which the Peptide Is Linked via a Cysteine-Based Unit to a Glycerol Moiety and One or More Lipid Chains Chiral carbon centers are indicated by * and **. Based on ref (48).

A number of lipopeptide adjuvants have been developed based on stimulation of TLRs. The core structure with one, two, or three lipid chains linked through a glyceryl cysteine linker to a peptide sequence is shown in Scheme 2, which shows the main peptide sequences that have been used and developed commercially. Peptide- and lipopeptide-based TLR agonist systems for vaccine development have been reviewed.^20,28,48,57,58^ Tripalmitoyl-*S*-glyceryl cysteine (Pam_3_Cys) is derived from the N-terminal moiety of Braun’s lipoprotein that spans the inner and outer membranes of Gram-negative bacteria. On the other hand, dipalmitoyl-*S*-glyceryl cysteine (Pam_2_Cys) corresponds to the lipid moiety of macrophage-activating lipopeptide 2 isolated from mycoplasma (bacteria which lack cell walls). It has also been reported that Pam_2_Cys is a more potent stimulator of splenocytes and macrophages than Pam_3_Cys.^59^ It also has a negligible pro-inflammatory response, as assessed in terms of cytokine (TNF-α, IL-1β, IL-6, and IL-8) release in a study using whole human blood.^60^ Pam_3_Cys activates TLR2 to produce a Th2 response, as evidenced by the production of associated effector molecules including IL-13 and IL-1β but not Th1-related cytokines.^61^ Other groups found that TLR2 stimulated by Pam_3_CSK_4_ promotes Th2 response,^62^ as well as that of Th17 which is implicated in several autoimmune conditions.^62−64^ The TLR2 activation was found to occur via other TLRs (TLR4 and TLR7/8), since cytokine production in human DCs induced by these TLRs was inhibited by TLR2.^62^ These reports seem to be contradicted by another study, in which it is reported that Pam_3_CSK_4_ and MALP-2 trigger Th1 cell function via TLR2 but do not stimulate any Th2 cell response.^65^ The X-ray crystal structure of the Pam_3_Cys ligand co-crystallized with the TLR1/2 dimer has been reported,^66^ as has the crystal structure of Pam_2_CSK_4_ with the TLR2/TLR6 dimer.^67^

In a pioneering paper, Braun reported that a lipoprotein component of the outer membrane of *E. coli* is a specific and potent antigen.^68^ This group had earlier identified Pam_3_CSSNAK as the N-terminal domain from the murein (peptidoglycan) lipoprotein obtained from the outer cell membrane of *E. coli*.^69,70^ Shorter fragments of the SSNAK peptide were also isolated,^70^ and synthetic lipopeptides bearing these sequences were prepared. All of them were found to be mitogenic and stimulated B lymphocytes into immunoglobulin secretion, as shown by a hemolytic plaque assay.^71,72^ The activity of all lipopeptides bearing different peptide fragments was comparable to that of the native *E. coli* lipoprotein, although those bearing only cysteine were hardly active.^72^ The Pam_3_CSSNAK lipopeptide was shown to also have adjuvant activity, as shown in a study of antibody response in sheep.^73^ This group also compared the activity of Pam_3_CSSNA, Pam_3_CSK_4_, Pam_3_CAG, and Pam_3_CS, and all lipopeptides were shown to have excellent adjuvant activity.^74^ The lipopeptide Pam_3_CSK_4_ was found to be a potent antigen able to fully replace Freund’s complete adjuvant (PCS) enhancing immunoglobulin production in mice.^74^ Earlier studies on Pam_3_Cys-based peptides with adjuvant or antibody-stimulating properties or self-adjuvating forms have been summarized.^75^

**Figure 2:**
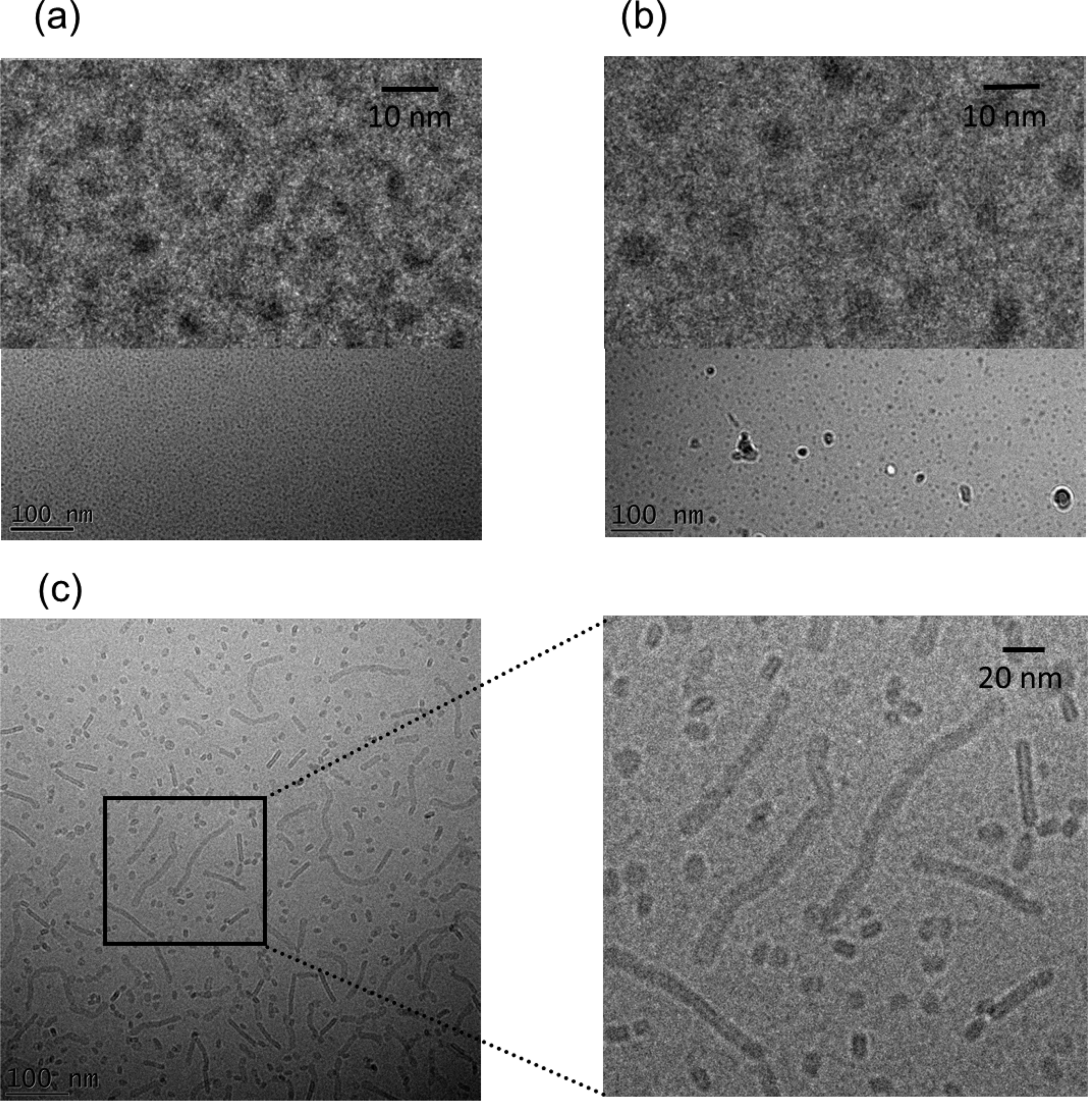
Cryo-TEM images of aqueous solution structures (2 wt % lipopeptide) of (a) PamCSK_4_, (b) Pam_2_CSK_4_, and (c) Pam_3_CSK_4_.^78^ Published by The Royal Society of Chemistry.

Peptides and lipopeptides such as Pam_2_CSK_4_ and Pam_3_CSK_4_ were developed with enhanced amphiphilicity (compared to the native *E. coli* murein lipoprotein), with the additional lysine residues conferring solubility.^74^ These molecules are agonists of TLR2 in particular and the Pam_3_Cys variants also with TLR1.^20,59,60,76,77^ The peptide conformation and self-assembly properties of the mono-, di-, and tri- palmitoylated CSK_4_ lipopeptides were compared using a combination of CD spectroscopy, cryo-TEM, and SAXS.^78^ This revealed that PamCSK_4_ and Pam_2_CSK_4_ form spherical micelles (Figure 2a,b) in which the peptide adopts an unordered conformation. In contrast, Pam_3_CSK_4_ forms a population of nanotape structures (Figure 2c), based on β-sheet aggregation (these disorder on heating).^78^ The experimental findings were confirmed by later atomistic molecular dynamics computer simulations.^79^

**Scheme 3:**
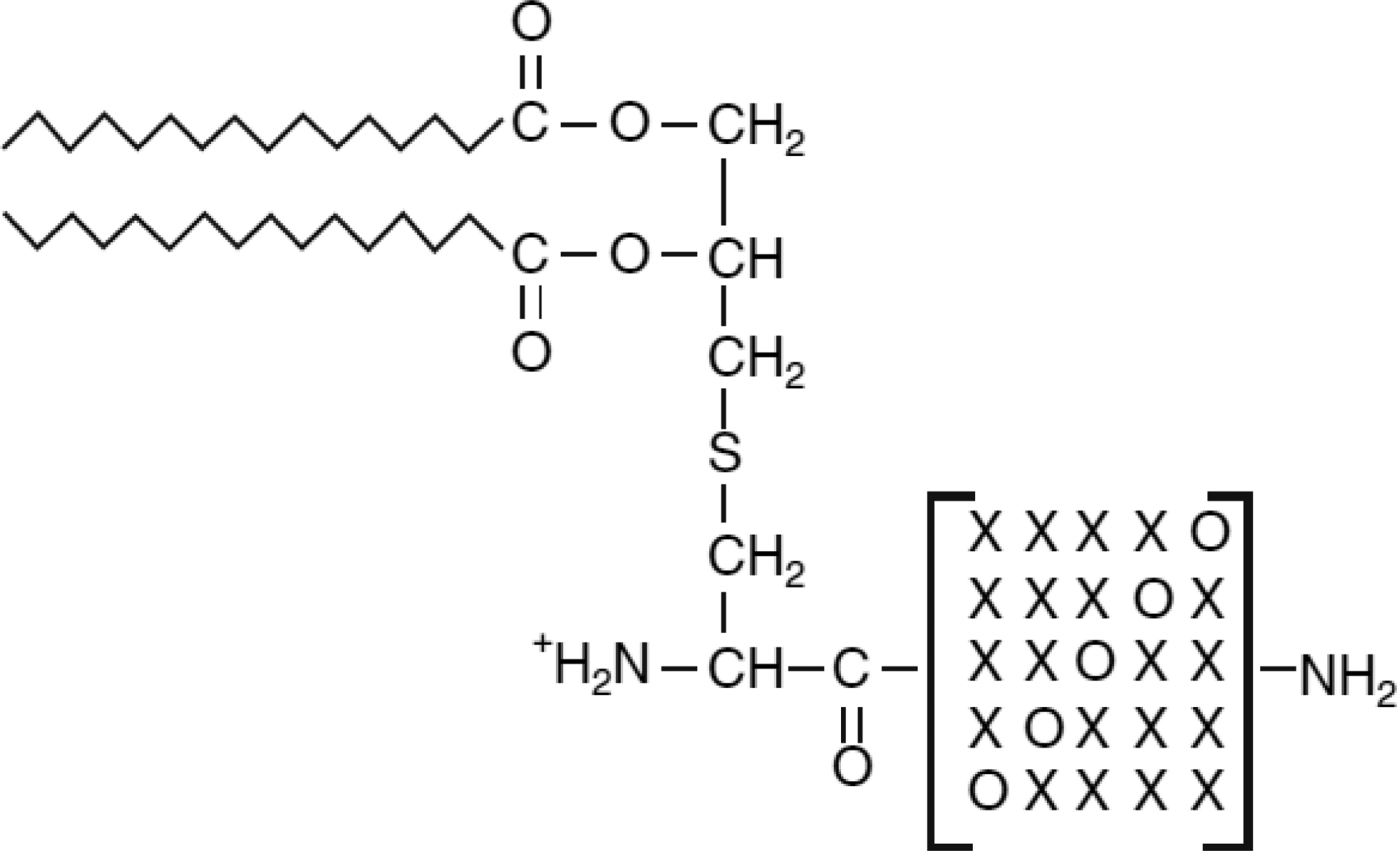
Pam_2_Cys Variants Studied by Wiesmüller and Coworkers Here X is one of the 20 standard amino acids and O is any of these amino acids except cysteine.^83^ Copyright 2005 John Wiley and Sons Inc.

Due to the potent immunogenic properties of Pam_*n*_Cys lipopeptides, much work has been undertaken to determine the structural features required for activity, as well as efforts to improve the response. Jung and co-workers prepared a range of analogues of Pam_2_CSK_4_ with variation in the length of the two alkyl chains and analogues of Pam_3_CSK_4_ with different *N*-acyl groups.^80^ They also determined the activity of the synthesized lipopeptides using HEK293 cells transfected with human TLR2 and firefly luciferase reporter gene, measuring the release of IL-8 in the culture medium and NF-κB translocation using luciferase reporter assay. They found that a critical length of more than eight carbons in the two ester chains is required to elicit a biological response, whereas the amide (*N*-acyl) chain length is less important.^80^ Triacylated lipopeptides with short ester-bound fatty acids such as PamOct_2_CSSNASK_4_ induce no response in TLR1-deficient cells,^81^ and Pam_2_CSK_4_ and the C-terminally elongated MALP-2 derivative Pam_2_CGNNDESNISFKEKSK_4_(MALP2-SK_4_) induce cell activation in a TLR6-independent manner.^82^ These molecules can be used as TLR-independent controls in studies of immunogenic response. This group also screened randomized Pam_2_Cys variants (Scheme 3) for B cell proliferation activity using murine splenic lymphocytes.^83^ This led to the identification of two lipopeptides, Pam_2_CGQHHM-NH_2_ and Pam_2_CSSHHM-NH_2_, with enhanced activity compared to Pam_3_CSK_4_. As well as the variants shown in Scheme 3, this group also investigated the effect of lipid chain length from C_6_–C_20_ in Pam-Cys(Dhp)-SSNASKKKK-based [Dhp: 3,4-dihydropyran] homologues, and the lipopeptide-induced IL-8 release from HEK 293-TLR2 cells was assayed.^84^ The activity was found to be significantly reduced for lipopeptides with lipid chain lengths shorter than C_10_. Ester-bound oleic acid and/or linoleic acid gave the best results in the IL-8 release assay.^84^

**Scheme 4:**
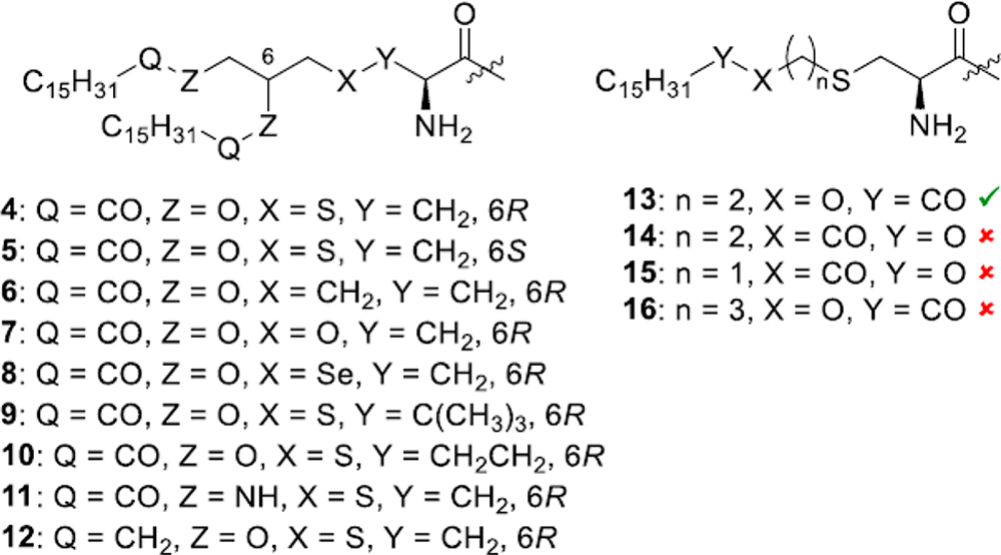
Summary of Molecular Structures from Structure–Activity Relationship Studies on TLR2 Agonist Lipopeptides Based on the *S*-(2,3-Dihydroxypropyl)-l-cysteine Core Present in Pam_2_CSK_4_ and PamCSK_4_^20^

As mentioned above, a number of groups have investigated structure–activity relationships in terms of the structure of the *S*-(2,3-dihydroxypropyl)-l-cysteine linker present in several TLR2 agonist lipopeptides. Scheme 4 summarizes the molecular structures in these studies.^20^

Studying MALP-2 (Scheme 2), Takeuchi et al. reported the significantly greater TLR2 activity of the *R* stereoisomer **4** (Scheme 4) compared to the *S* version **5**, based on the analysis of cytokines, chemokines, or NO released by cells.^85^ Substitution of the thioether moiety with either a methylene (**6**)^86,87^ or oxyether (**7**)^88^ group leads to a loss in TLR2 activity, whereas substitution of S with Se (in **8**) causes an upregulation of p38 mitogen-activated kinase (p38MAPK) in neutrophils.^89^ The *R*-cysteinyl form of Pam_2_CSK_4_ is also maximally active in reporter gene assays using human TLR2, as revealed in a study of the role of the highly conserved Cys residue as well as the geometry and stereochemistry of the Cys-Ser dipeptide unit.^88^

Changing the length of the spacer between the thioether and the α-center of cysteine (**9** containing a penicillamine-based linker or homocysteine-based **10**) leads to dramatic reductions in TLR2 activity.^89^ Substitution of the ester functionalities for amide (in **11**)^90^ or ether (in **12**)^91^ groups in lanthionine scaffolds strongly inhibits TLR2 activity. Compound **13** (corresponding to PamCys, Scheme 2) has pronounced TLR2 agonist activity.^89^ However, inversion of the ester bond (in **14** and **15**) or extension of the chain between the thioether and ester groups leads (in **16**) to a significant reduction in activity.^89^ A derivative with a thioethanol bridge in place of the thiogylcerol unit retained TLR2-specific NF-κB induction activity.^89^ The role of stereochemistry (*R*- and *S*-stereoisomers of the glycerol moiety) has been examined for Pam_3_CSK_4_, and it was found that the *R*-stereoisomer has greater activity,^75,92^ similar to the case of MALP-2. This was ascribed to enhanced TLR2 triggering due to greater DC activation which stimulates CD8^+^ T cell responses, in particular higher IL-12 secretion, and upregulates relevant markers for DC maturation.^92^ Methylene substitution [to give 2-(palmitoylamino)-6,7-bis(palmitoyloxy)heptanoic acid) (**6**)] leads to lower activity than Pam_3_CSK_4_ at a given concentration, although the maximal activity obtained at significantly higher concentration is similar to that of Pam_3_CSK_4_.^87^ The role of stereochemistry was also examined, all four stereoisomers being prepared, the 6*S* form was active in terms of radiolabeled [^3^H]thymidine incorporation (a cell proliferation assay) into the DNA of mice splenocytes after stimulation with lipopeptides than the 6*R* form, with little influence of the other (2*S*/2*R*) stereocenter.^87^ The authors also noted that these lipopeptides constitute potent macrophage activators with anticancer activity: in particular, the (2*S*,6*S*) stereoisomeric form was able to induce tumor cytotoxicity.^87^ Further details of the synthesis of the derivatives discussed in this section are provided by Brimble and co-workers.^20,58^

**Scheme 5:**
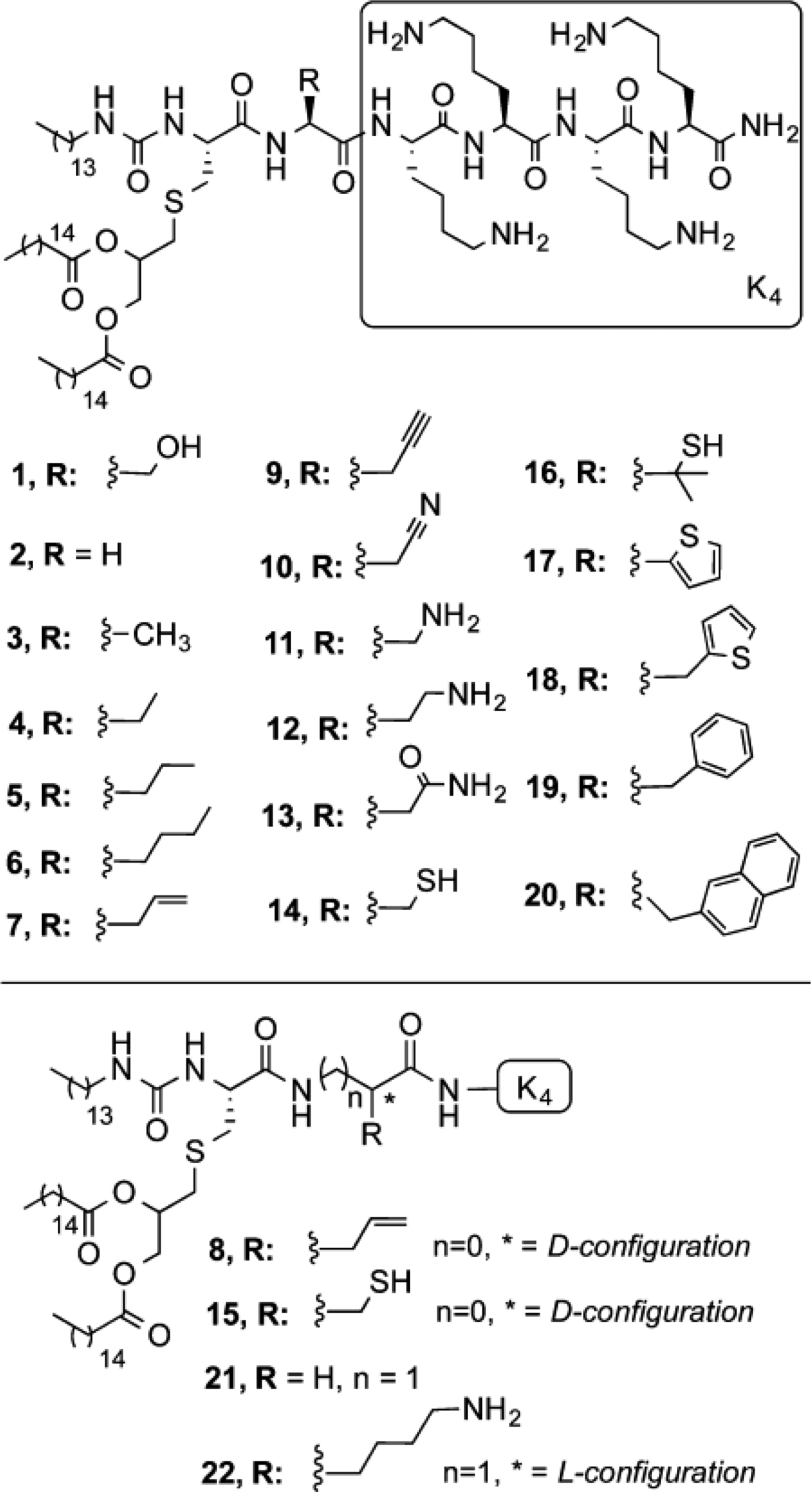
Urea-Functionalized Pam2Cys Analogues (UPam Derivatives) Studied by Willems et al.^93^

A series of Pam_2_CSK_4_ analogues (Scheme 5) were prepared that contain a urea (carbamyl) linker (hence, they were termed UPam) to one lipid chain and a range of substitutions for the serine residue.^93^ The motivation for the *N*-tetradecylcarbamyl substitution was the expectation that immunostimulatory activity would be enhanced, on the basis of modeling of the crystal structure of the X-ray structure of the Pam_3_Cys ligand co-crystallized with the TLR1/2 dimer.^66^ Probing DC maturation, the authors observed that UPam derivatives **3**–**5**, **7**, **9**, and **10** (Scheme 5), which contain small, mostly hydrophobic side chains substituting for the hydroxymethyl of **1** (UPam-Ser), show an increased potency compared to Pam_3_CSK_4_ and those containing amino acids with straight alkyl chains of moderate size terminated with a polar functional group (**12** and **13**) also have enhanced activity (compared to Pam_3_CSK_4_). In contrast, the derivatives **8**, **14**, **15**, **20**, and **22** were found to have minimal activity.^93^

**Scheme 6:**
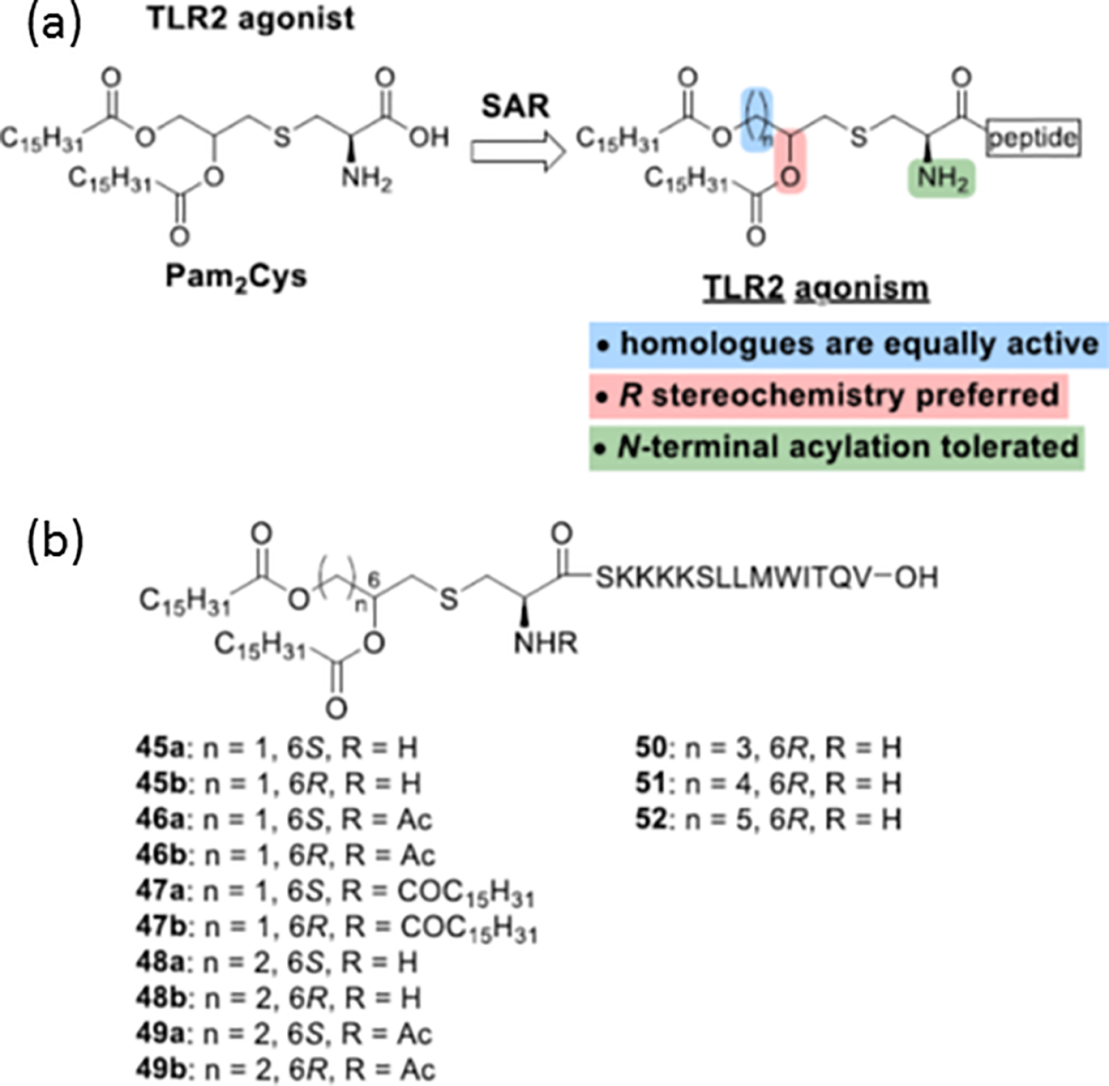
Pam_2_CSK_4_-Based Lipopeptide Molecular Structures Studied by Brimble’s Group^20^ (a) Schematic of the architecture, (b) molecular structures of specific molecules investigated.

Lipopeptide Pam_3_CSK_4_ has been investigated as an adjuvant. For example, it can elicit immunogenicity, i.e., CTL responses to *Plasmodium berghei* circumsporozoite (CS, a parasite that can cause malaria in rodents) peptide epitope SYIPSAEKI either in mixtures or by conjugation of this sequence onto Pam_3_CSK_4_ (to give Pam_3_CSK_4_SYIPSAEKI), as assayed using mouse splenocytes.^94^ In addition, a conjugate of Pam_3_Cys with poly(ethylene glycol) (PEG) also known as polyoxyethylene (the synthesis of the conjugate being described earlier^95^) was investigated as part of the range of formulations studied, and this conjugate mixed with CS also generated a CTL response.^94^ The influence of the structure of Pam_2_Cys analogues on TLR2 agonist activity prepared as potential adjuvants for cancer vaccines has been examined.^20^ The effects of homologation between the two ester functionalities, *N*-terminal acylation, and acyl group stereochemistry (Scheme 6) were examined in lipopeptides bearing the SK_4_ sequence linked to an epitope from the tumor associated NY-ESO-1 protein based on the sequence SLLMWITQC (Scheme 6b).^20^ For the bioactivity assays, HEK-Blue-hTLR2 cells were transfected with the TLR2-NF-κB-SEAP reporter-gene system. This system has been taken forward into commercial development by SapVax.^96^ Pam_3_CSK_4_ has also been used in the development of an HIV vaccine, by conjugation to the 32-amino-acid group-specific antigen peptide containing at least five CTL epitopes, NPPIPVGEIYKRWIILGLNKIVRMYSPTSILD.^97^

**Scheme 7:**
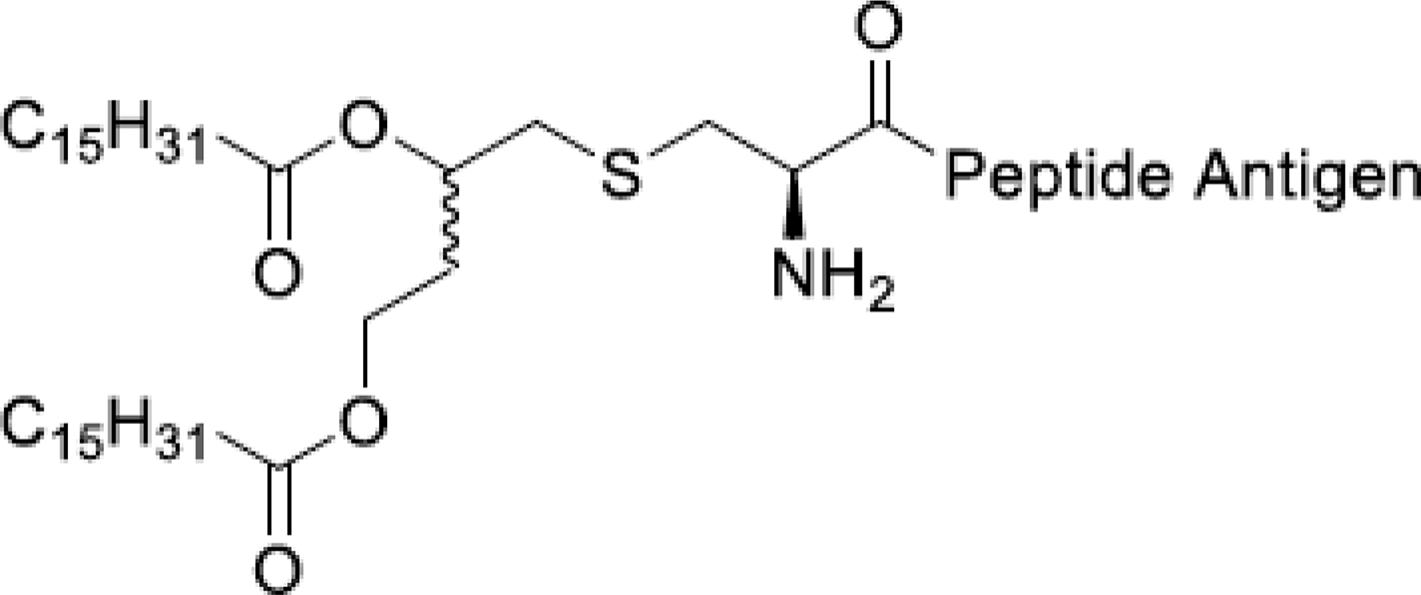
Homologue of Pam_2_Cys Termed *homo*Pam2Cys Prepared by Brimble’s Group^20^

Direct thiol–ene coupling has been used to alkylate CSK_4_ with an *S*-palmitoyl chain at the N-terminus.^98,99^ In addition, as a model vaccine cytomegalovirus (CMV) ppUL83 protein sequence NLVPMVATV^100^ was attached to produce an antigenic lipopeptide bearing the SK_4_NLVPMVATV sequence. Strong up-regulation of the co-stimulatory molecule CD80 on human monocytes in fresh blood samples was observed using flow cytometry, higher than that for Pam_3_CSK_4_.^98,99^ This reaction was later studied in more detail, due to the presence of an observed byproduct (shown in Scheme 7), and conditions to obtain either mono- or bis-palmitoylated cysteine derivatives were identified.^101^ The molecule in Scheme 7 is a homologue of Pam_2_Cys with an extra methyl group in the glyceryl linker and was termed *homo*Pam2Cys. It was isolated as a mixture of stereoisomers and was tested for NF-κB induction, and it showed the same activity as Pam_2_Cys itself.^20^ The effect of the stereochemistry was investigated by preparing *R* and *S* enantiomers of Pam_2_Cys, *homo*Pam2Cys, and analogues.^20^ Based on the NY-ESO-1 protein sequence SLLMWITQC mentioned above and the HEK-Blue TLR2 reporter system, it was found that the *R* diastereomers have activity, whereas the *S* versions show a pronounced reduction in TLR2 activity.^20^

**Scheme 8:**
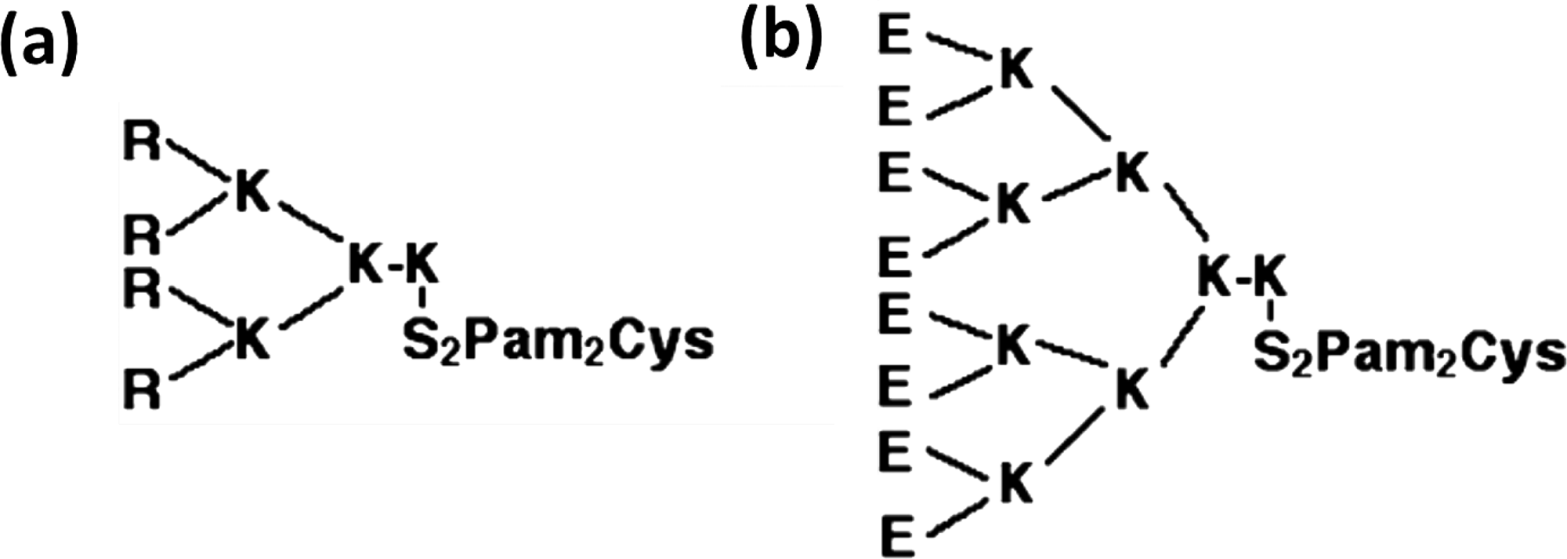
(a) R_4_Pam_2_Cys, (b) E_8_Pam_2_Cys^102^ Originally published in *The Journal of Immunology*. Copyright 2011 The American Association of Immunologists, Inc.

**Scheme 9:**
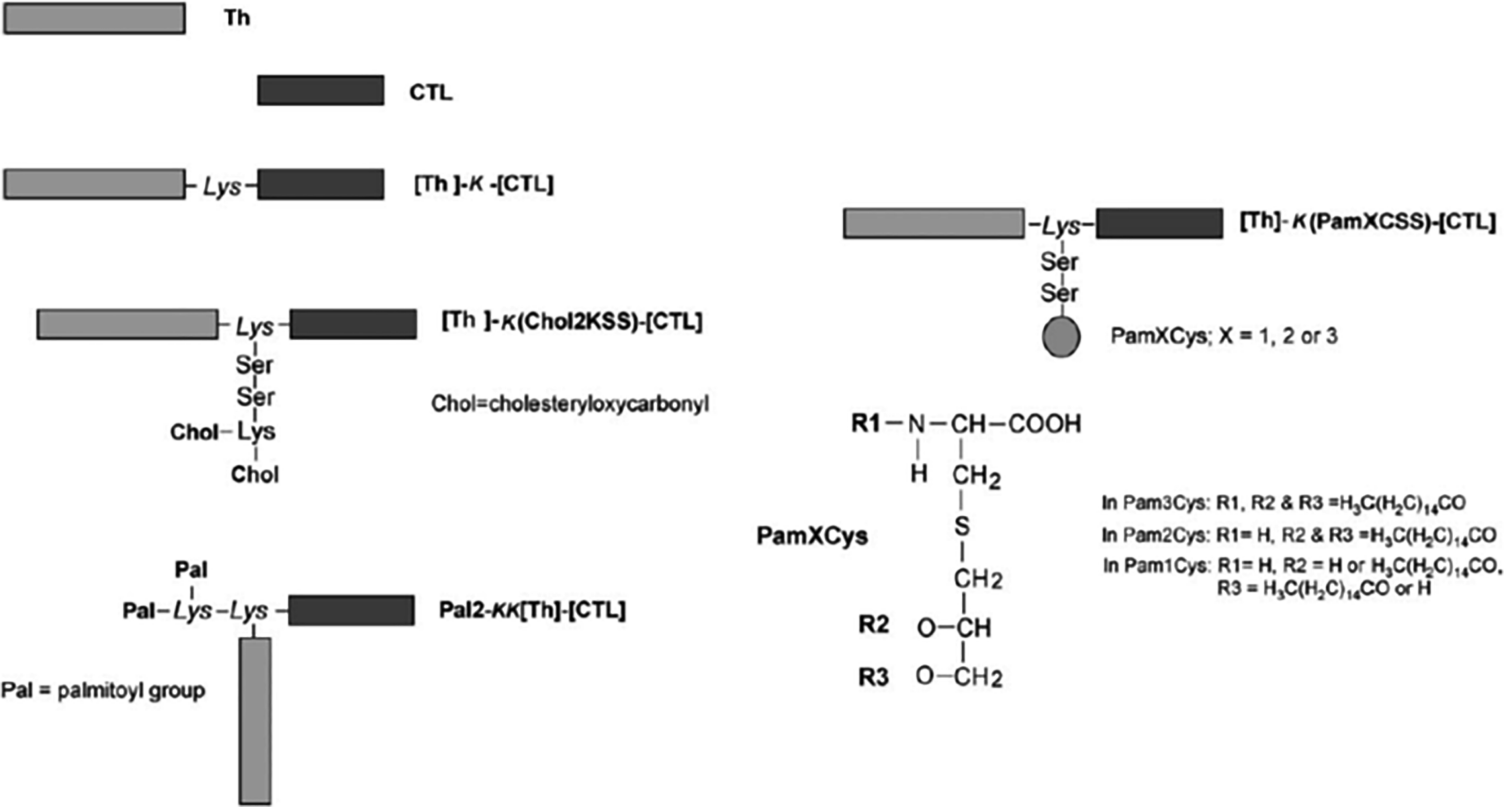
Architectures of Dual-Action Th- and CTL-Containing Lipopeptide Constructs Bearing Pam_*n*_Cys Units Studied by Jackson’s Group^105^ Reprinted by permission from Oxford University Press.

Derivatives based on Pam_2_Cys bearing branched highly cationic or anionic N-termini have been designed (Scheme 8) in order to facilitate interaction with oppositely charged soluble protein antigens.^102,103^ The binding of lipopeptide to oppositely charged antigens leads to the formation of complexes at physiological pH.^102^ The complexes elicit a CD8^+^ T cell immune response in mice, with concomitant pro-inflammatory cytokine production. Model antigens selected were ovalbumin (OVA) or hen egg lysozyme (HEL) with a net calculated charge of −11 or +8, respectively. The complexes were also shown to protect vaccinated mice against challenge with a live chimeric influenza virus engineered to contain the OVA CTL epitope SIINFEKL. The induced CD8^+^ T cell response correlated with the ability of lipopeptide to facilitate antigen uptake by dendritic cells (DCs). Oppositely charged lipopeptides were more effective in DC uptake and trafficking. Substantial antibody titers were produced by vaccination with complexes composed of oppositely charged lipopeptide and protein, whereas innoculation with similarly charged constituents resulted in measurable but lower antibody production.^102^ Peptide R_4_Pam_2_Cys (Scheme 8) shows a strong ability to bind OVA, leading to the formation of large complexes (ca. 400 nm radius from DLS).^103^ Excellent adjuvant activity was noted, from measurements of antibodies in mice and cytokine production. Antibody titers were higher for the lipopeptide/OVA mixture than those elicited by OVA in the presence of alum and were comparable to those elicited by OVA formulated with complete Freund’s adjuvant (CFA). The activity of the d-Arg homologue of R_4_Pam_2_Cys was also examined and was shown to stimulate similar levels of antibody production, although CD8^+^ T cell responses (IFN-γ secretion) were reduced.^103^ Covalent conjugates of Pam_3_CSK_4_ with OVA_247–264_ peptide epitope DEVSGLEQLESIINFEKL (or another peptide with this sequence and an A_5_K C-terminal extension) were prepared in a study of the TLR2 processing.^104^ It was found that the uptake of the conjugates is TLR-independent, and inhibition of clathrin- or caveolin-dependent endocytosis greatly reduced uptake and antigen presentation of the Pam_3_CSK_4_ conjugates. The lipopeptides induce a strong and specific T cell response due to the combined effects of enhanced antigen uptake, improved MHC class I antigen presentation, and dendritic cell maturation.^104^ The Pam_2_Cys scaffold has been employed in the development of a range of immunogenic lipopeptides. In one example focused on the development of self-adjuvanting immunocontraceptive vaccines, Pam_2_Cys linkers (incorporated via lysine ε-amino units) were compared to Pam_3_Cys and control sequences just bearing lysine linkers, or other constructs with only C-terminal peptide sequences.^59^ These bifunctional lipopeptides bear both the LHRH (luteinizing hormone releasing hormone) sequence EHWSYGLRPG and a T-helper epitope GALNNRFQIKGVELKS (from the L chain of influenza virus hemagglutinin). The lipopeptides induce antibodies against the “self” hormone LHRH in inoculated mice, without requiring additional adjuvant. The Pam_2_CSS lipopeptide attached laterally via a central K residue linking the two epitopes was highly effective, inducing high antibody titers, which were able to efficiently sterilize female mice when administered in saline by s.c. or intranasal routes.^59^ The architecture with pendant Pam_*n*_CSS linked via the ε-amine group of lysine (Scheme 9) is also most effective in stimulating DC maturation via TLR2.^105^ This was confirmed in a study using lipopeptides with this architecture bearing a Th sequence ALNNRFQIKGVELKS from influenza hemeagglutinin that elicits CD4^+^ T cells and a CTL peptide epitope TYQRTRALV, i.e., NP_147–155_, derived from the nucleoprotein of the influenza virus which is the dominant CD8+ T cell epitope recognized by BALB/c mice in all type A influenza strains.^105^

**Scheme 10:**
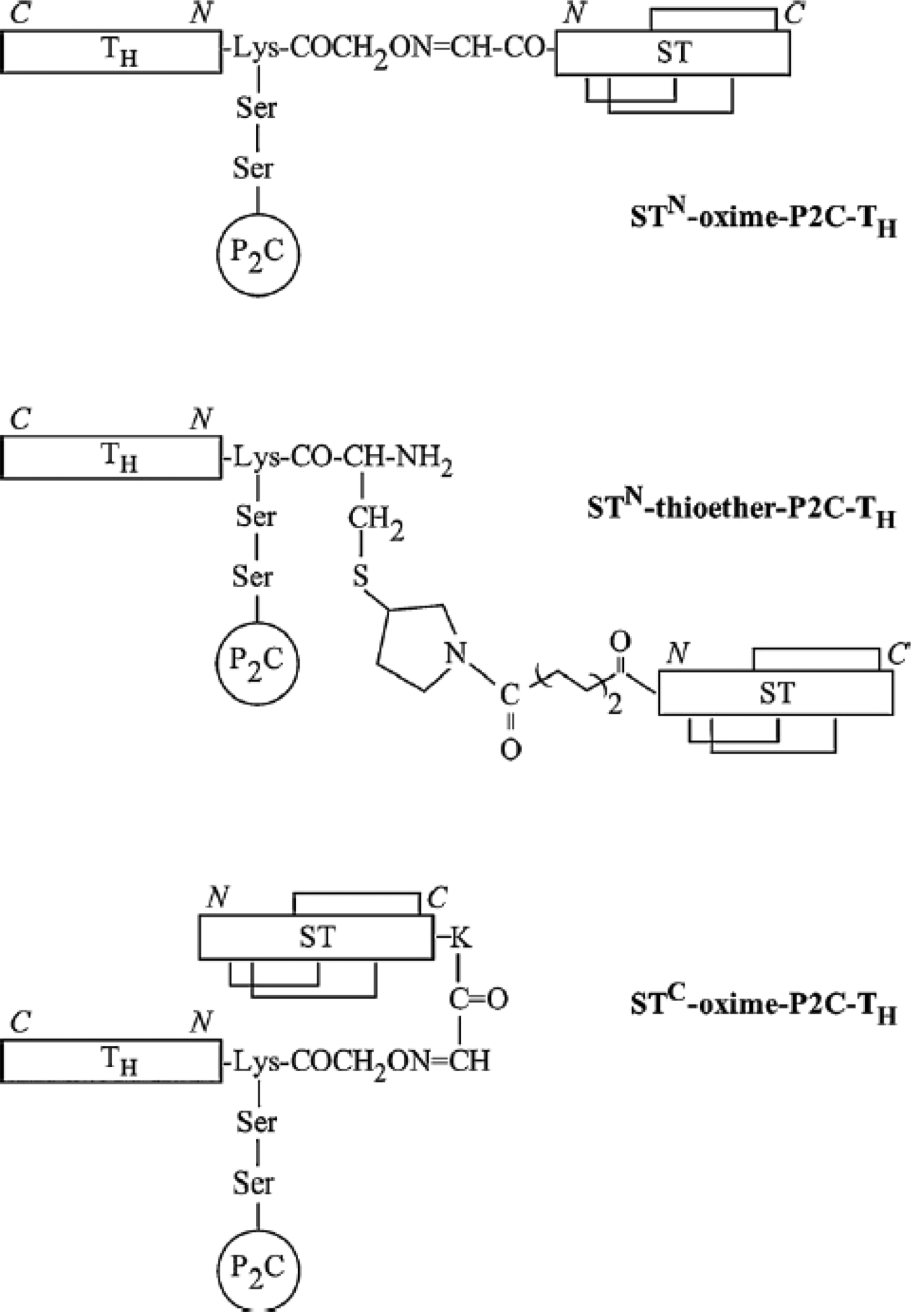
Lipopeptide Conjugates Prepared by the Jackson Group with Different Linkers between a Helper T Cell Domain (Th) and the Heat-Stable Enterotoxin ST Domain The Pam_2_Cys (P_2_C) unit is attached laterally via a KSS linker. Reprinted from ref (106) with permission. Copyright 2012 Elsevier.

In another example, bifunctional Pam_2_Cys-based derivatives were designed as a self-adjuvanting vaccine that induces neutralizing antibodies against heat-stable enterotoxin from enterotoxigenic *E. coli*.^106^ Three constructs were synthesized with an N-terminal helper T cell epitope (GALNNRFQIKGVELKS, as above) and a C-terminal heat-stable enterotoxin (ST) tricylic peptide^107^ NSSNYCCELCCNPACTGCY (C6–C11, C7–16, and C10–C18 disulfide links) with different linker groups to the ST peptide (Scheme 10). All three compounds generated specific anti-ST antibodies; however, the low titer antibodies (using a mouse model) induced by the oxime-containing derivative demonstrated better neutralizing activity when administered via the intranasal mucosal route.^106^ As pointed out by Moyle and Toth,^48^ the delivery of antigens mixed with, or conjugated to, lipopeptide adjuvants has the potential to produce vaccines that are immunogenic via mucosal routes, in particular nasal or oral.

The Jackson group showed that in fact this modular approach can be extended to produce immunogenic lipopeptides bearing LHRH or acid polymerase (PA) of influenza virus with sequence SSLENFRAYV or the ST enterotoxin mentioned above linked by oxime, thioether, or disulfide units to one of three T helper cell motifs.^108^ The first was T_h_(OVA) derived from OVA, with sequence ISQAVHAAHAEINEAGR, the second was T_h_(FLU), which is derived from the light chain of influenza virus hemagglutinin (sequence ALNNRFQIKGVELKS), and the third was T_h_(MV), derived from the fusion protein of the *Morbillivirus* canine distemper virus (sequence KLIPNASLIENCTKAEL). The authors note that using a thioether bond to conjugate the two components has the advantage of simpler chemistry and somewhat higher yield of product, whereas disulfide or oxime bond formation requires an additional synthesis step. Oxime bond formation, however, does have the advantage that the unwanted side products resulting from thiol groups present in cysteine residues (if present) may be avoided. The antibody and CD8^+^ T cell responses were found to depend on both the linker group and the Th epitope employed.^108^ In a related study, the Th sequence LNNRFQIKGVELK derived from the light chain of influenza hemagglutinin was linked via the same linker with appended Pam_2_CSS (Scheme 9) to the CTL epitope, with sequence TYQRTRALV, mentioned above.^109^ The Th domain was selected because it elicits CD4^+^ T cells that are cross-reactive with all H3 influenza viruses and the CTL (CD8^+^) epitope is common to all type A influenza strains. This lipopeptide can be delivered intranasally and is able to prime lung-resident memory CD8^+^ T cells for long-term pulmonary protection against influenza.^109^ In another case, three Pam_2_Cys-based conjugates were prepared which contain a solubilizing spacer composed of either sequences of lysine residues or polyethylene glycol (PEG).^110^ The model proteins HEL or bovine insulin were lipidated with the three types of lipid-spacer moieties, and the immunogenicity of the lipidated species was determined in mice by measuring antibody responses. The lipopeptides contain C-terminal cysteine residues which allow for formation of either a thioether or a disulfide bond with proteins (or peptides) derivatized with MCS [*N*-(ε-maleimidocaproyloxy acid succinimide ester] or SPDP [succinimidyl 3-(2-pyridyldithio)propionate], respectively.^110^ The Pam_2_Cys scaffold has been used to attach both helper T cell Th and a target epitope that is either recognized by CD8^+^ T cells or B cells, using a diversity of Th and target epitopes.^111^ These examples highlight research showing that the modular (“subunit”) approach to the self-adjuvant vaccine development offers considerable scope to tune the immune response. A review is available that details methods to synthesize the lipopeptides designed by the Jackson group.^112^

The Pam_3_CSS scaffold has been used along with the 16 N-terminal amino acids of the *M. tuberculosis* 19 kDa lipoprotein to give Pam_3_CSSNKSTTGSGETTTA which has been shown to be an agonist of RP105, a member of the TLR family that interacts with TLR2 and facilitates recognition of mature lipoproteins expressed by mycobacteria.^113^ Mono- and dipalmityol analogues were also examined, as were peptide variants. These studies showed that, although the lipid moiety is required for macrophage activation, it is not a determinant of RP105 dependency. However, substitution of the T7 and T8 residues with nonpolar alanine residues led to reduced RP105 dependency.^113^

Like Pam_3_CSK_4_, the *M. tuberculosis* 19 kDa lipoprotein (lipopeptide) stimulates T cell proliferation via TLR2 activity, producing IFN-γ in an accessory cell-dependent manner. Sieling et al. compared the activities of these two lipopeptides along with the *Treponema pallidum* lipoprotein TP47.^76^*Treponema pallidum* and *Borrelia burgdorferi* are the spirochetal pathogens responsible for syphilis and Lyme disease, respectively, and lipoproteins from the membranes of these organisms elicit a strong immunoinflammatory response, as potent activators of monocytes/macrophages.^114,115^ The OspA outer surface protein from *B. burgderfori* contains a post-translational amino-terminal Pam_3_Cys lipid moiety, and this component is essential in producing an antibody response.^116^ The full OspA lipoprotein showed significantly better adjuvant activity than Pam_3_CSK_4_ in a study of intranasal delivery. Synthetic lipohexapeptides corresponding to the N termini of the *B. burgdorferi* strain B31 OspA lipoprotein (CKQNVS) and the *T. pallidum* subspecies *pallidum* 47-kDa lipoprotein (CGSSHH) prepared as glyceryl cysteine derivatives (with a Pam_3_C motif) show similar activity, as does the unlipidated OspA hexapeptide.^115^ The binding domains of the p19 *M. tuberculosis* 19 kDa lipoprotein to MHC class I binding motifs H-2D^b^ and H-2L^d^ have been identified.^117^ Several samples from a series of PamCSS-based lipopeptides investigated elicit a CTL response, assayed as IFN-γ production from mouse splenocytes; in particular, lipopeptides which upregulated MHC-I molecules (with a H-2D^b^ binding motif) showed this activity.^117^

Self-adjuvant breast cancer vaccines based on Pam_3_CSK_4_ were prepared using the HER2 (human epidermal growth factor receptor-2) epitope CH401, YQDTILWKDIFHKNNQLALT.^118^ Other formulations included a lipopeptide based on this sequence in a single palmitoylated peptide with a diethylene glycol linker and mixtures of this with Pam_3_CSK_4_ or the sphingolipid α-GalCer (a strong immunostimulant with antitumor properties) or Lipid A or a Pam-Th epitope QYIKANSKFIGITE (tetanus toxin-derived epitope). The conjugate self-adjuvanting vaccine produced a stronger antibody response (IgG titers from mice) than that of Pam_3_CSK_4_ itself. The conjugate Pam_3_CKS_4_–CH401 conjugate self-assembles into globular aggregates, as revealed by TEM, whereas mixtures of PamCSK_4_ and Pam-CH401 showed fibrillar co-assemblies, also observed for other mixtures containing Pam-CH401. The co-assembled structures also elicited a robust immune response.^118^

The conformational and self-assembly properties of MALP-2 have been examined in detail.^119^ MALP-2 is a macrophage-activating lipopeptide (hence the name) originally isolated from *Mycoplasma fermentas* and contains a 13-residue peptide GNNDESNISFKEK attached to a Pam2Cys linker (Scheme 2).^120^ Whereas three lipid chain lipopeptides such as Pam_3_CSK_4_ signal via TLR1 and TLR2 as discussed above,^20,49,59,60,76,77^ MALP-2, in common with other two-lipid-chain lipopeptides,^28^ signals via TLR2/TLR6 heterodimers with CD36 as co-receptor.^77,121,122^ Both MALP-2 and the constituent peptide adopt β-sheet conformations with different morphologies, with MALP-2 forming fibril rafts, whereas the peptide alone forms nanotape structures.^119^ Pro-inflammatory responses in mouse lungs induced by MALP-2 and Pam_3_CSK_4_ have been compared, considering the fact that the lungs have the highest expression of TLR2 receptors. The authors observed that the MALP-2-dependent induction of Tnc (tenascin C, a glycoprotein involved in inflammation) may indicate the existence of TLR2/6-specific pathways.^77^ The recognition of MALP-2 by TLR6 cooperatively with TLR2 was noted, together with the observation that TLR6 appears to discriminate between the N-terminal lipidated structures of MALP-2 and Pam_3_CSK_4_.^121^ Indeed, by comparing MALP-2 with the variant of MALP-2 containing three rather than two palmitoyl chains, it was inferred that three-chain lipopeptides are recognized by TLR2 while two-chain molecules (MALP-2 or lipoteichoic acid) require additional cooperation with TLR6.^122^ In subsequent work, Mühlradt’s group compared the macrophage-stimulating activities of two lipopeptides isolated from *Mycoplasma hyorhinis* and several synthetic peptides including Pam_2_CSK_4_ and Pam_3_CSK_4_ with MALP-2.^123^ The assays measured nitric oxide release assay with peritoneal macrophages from C3H/HeJ mice. The isolated peptides were analogues of MALP-2 containing (in some cases) different lipid chains (and in all cases different peptide sequences), i.e., *S*-[2,3-bisacyl(C16:0/C18:0)oxypropyl]cysteinyl-GQTDNNSSQSQQPGSGTTNT and *S*-[2,3-bisacyl (C16:0/C18:0)oxypropyl]cysteinyl-GQTN and an additional synthetic peptide *S*-[2,3-bis(palmitoyloxy)propyl]cysteinyl-GQTNT was examined. The GQTNT motif is from the determined variable lipopeptide C (VlpC) sequence. The lipopeptides showed pM activity, i.e., were as active as MALP-2, except for Pam_3_CSK_4_ which showed activity above 100 pM.^123^ A PEGylated version of MALP-2 has been shown to produce strong humoral and cellular immune responses against enterohemorragic *E. coli* after intranasal vaccination in a mixture with enterohemorragic *E. coli* antigens.^124^ The MALP-2 derivative was effective as an adjuvant in this mucosal (nasal) vaccine study due to its improved solubility. MALP-2 has also been used in combination with gemcitabine (a chemotherapy medication) in a phase I/II clinical trial of an immunotherapy for patients with pancreatic carcinoma.^15^ Lipopeptides have also been derived from the 44 kDa membrane-bound lipoprotein of *Mycoplasma salivarium*, including the N-terminal lipopeptide *S*-(2,3-bisacyloxypropyl)-cysteine-GDPKHPKSFTEWVA-, which was used as the basis to create the synthetic analogue *S*-(2,3-bispalmitoyloxypropyl)-cysteine-GDPKHPKSF.^125,126^ The latter is commercially available as FSL-1 (fibroblast-stimulating lipopeptide)^126^ and, like other Mycoplasma derivatives such as MALP-2, is recognized by TLR2 and TLR6.^127^ Variants of FSL-1 with a substitution of the C-terminal F for R or substitution of the palmitoyl lipid chains for stearoyl chains were also reported.^127^

The fact that TLR2/6 agonists can reduce virus levels in the upper respiratory tract has motivated the recent investigation of the use of a Pam_2_Cys analogue in the treatment of COVID-19 caused by the SARS-Cov-2 virus.^128^ Intranasal prophylactic administration of a compound INNA-051, a PEGylated Pam_2_Cys analogue (structure not disclosed but related to that shown in ref (110)), was shown to reduce SARS-CoV-2 transmission and to provide protection against COVID-19. Stimulation of TLR2 leads to activation of the innate immune response, reduced inflammation and tissue damage, and improved local epithelial barrier function.^128^ However, investigating respiratory syncytial virus infections, Nguyen et al. concluded that modulation of infection using Pam_3_CSK_4_ is independent of TLR activation.^129^ This was based on the observation that two structurally related lipopeptides (PamCSK_4_ and PHCSK, a negative control for Pam_3_CSK_4_ lacking a methylene group in the thioether spacer) without TLR-signaling capacity did modulate RSV infection, whereas Pam_3_CSK_4_-based TLR1/2 agonists did not.^129^ Studying COVID-19 disease severity and its relationship to TLR2 signaling, Zheng et al. identified an optimal TLR2 inhibitor by confirming the effectiveness and specificity of two different inhibitors of TLR2 signaling in bone-marrow-derived macrophage stimulated with Pam_3_CSK_4_.^130^ Thus, Pam_3_CSK4 has utility as a model agonist in studies on TLR2 inhibitors for COVID-19 vaccine development.

**Scheme 11:**
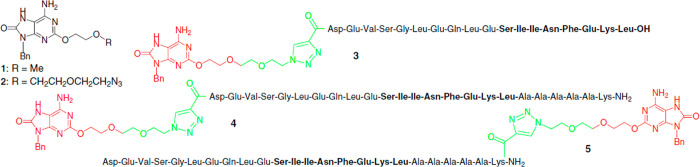
Conjugates of a 2-Alkoxy-8-hydroxyadenyl Derivative (**1**) with Peptides (**3**–**5**) Prepared by Weterings et al.^131^ In the conjugates, the 2-alkoxy-8-hydroxy adenine group is shown in red, while green indicates the linker region resulting from the Cu-catalyzed click cycloaddition reaction (via the azide unit in **2**). The OVA peptide sequence SIINFEKL is shown in bold. Reprinted from ref (131) with permission. Copyright 2006 Elsevier.

Conjugates of peptides with nucleotides have been prepared as TLR-dependent immunogens. In one example, the 2-alkoxy-8-hydroxyadenylpeptide conjugates shown in Scheme 11 were prepared.^131^ The nucleotide component is believed to act as a TLR7 ligand. These conjugates bear the MHC class I epitope SIINFEKL from OVA. In comparison with a mixture of their individual components, these conjugates gave rise to enhanced antigen presentation *in vitro* but were found not to be able to induce DC activation.

## 5 Lipid Core Peptides

**Scheme 12:**
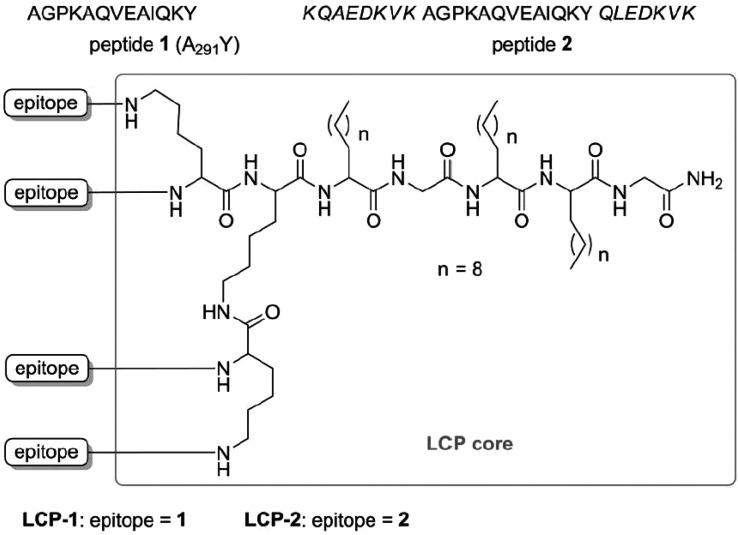
Structure of Lipid Core Peptide (LCP) Derivatives Developed as a Peptide-Based Subunit Vaccine against Hookworm Infection^133^

Toth’s group has developed so-called lipid core peptide (LCP) systems to deliver peptide epitopes at high density for a variety of applications, including the development of self-adjuvant vaccines.^48,132^Scheme 12 shows an example from a study that aimed to develop a peptide-subunit-based vaccine for hookworm infection.^133^ The LCP in this example comprises three lipidic amino acid chains and a four-arm amine functionalized dendrimer. The constructs prepared bear one of two peptide epitopes shown in Scheme 12. One is a B cell peptide epitope from the apical enzyme in the hemoglobin digestion cascade, the aspartic protease Na-APR-1 termed A291Y (peptide **1**, Scheme 12), while chimeric peptide **2** contains the A_291_Y sequence flanked on either side by helix-promoting sequences from the yeast GCN4 protein. It was found that, while A291Y alone or the chimeric peptide with or without Freund’s adjuvants induce negligible antibody responses, the LCP construct incorporating the chimeric peptide induces a strong IgG response in mice. The active chimeric peptide was designed to induce the native helical A291Y epitope conformation.^133^

**Scheme 13:**
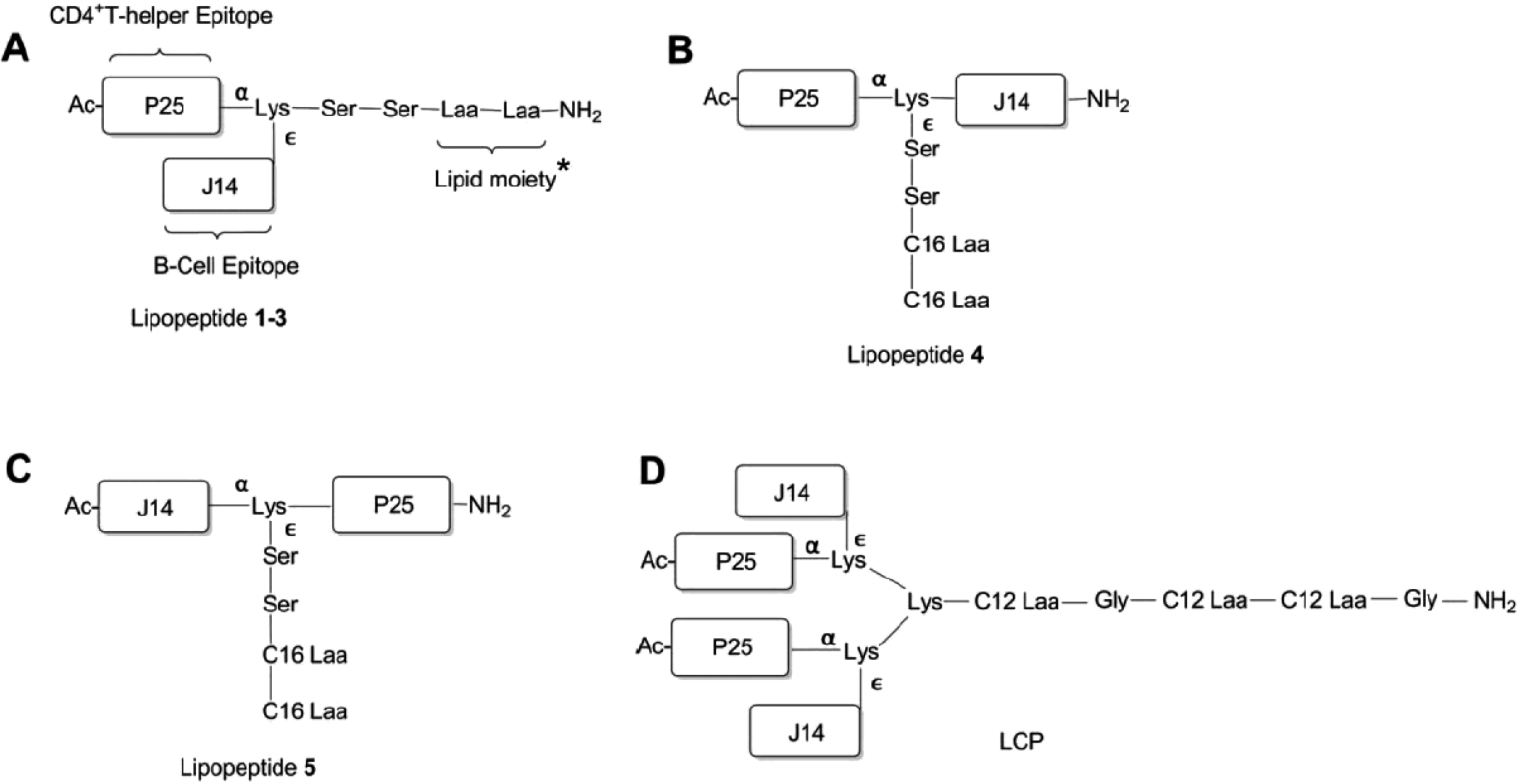
(A–C) Lipopeptide and (D) LCP Constructs Based on Lipoamino Acids (LAAs) and the J14 and P25 Peptide Epitopes Discussed in the Text^137^

This group also developed lipopeptide-based conjugates in an effort to produce a Group A Streptococcus (GAS) vaccine.^134−137^ The architectures of other constructs prepared by this group are shown in Scheme 13, and all contain the J14 peptide epitope KQAEKVK**ASREAKKQVEKALE**QLEDKVK, in which the 14-residue peptide GAS M-protein C region (B cell epitope) is shown in bold, along with a universal helper T cell epitope KLIPNASLIENCTKAEL (P25). The LCP **D** in Scheme 13 contains two copies of J14 and P25. The vaccine of type **A** (Scheme 13) featuring a C-terminal palmitoyl lipid moiety, with P25 located at the N-terminus, and J14 attached to the side chain of a central lysine residue was capable of inducing the optimal antibody response after intranasal immunization in mice.^134,137^ This group had earlier developed a LPC bearing the 88/30 GAS sequence DNGKAIYERARERALQELGC and the J8 GAS peptide sequence QAEDKVK**QSREAKKQVEKALK**QLEDKVQ (with bold core sequence related to that in J14) in the development of a self-adjuvant intranasal vaccine targeting the GAS M protein.^138,139^ The LCP vaccine formulation induced the elicitation of antigen-specific IgG production when administered with or without mucosal adjuvant cholera toxin B subunit, whereas cholera toxin B subunit was required for the induction of antigens. This group also developed human papilloma virus (HPV) vaccines based on an LCP bearing four copies of a 19 amino acid long sequence QAEPDRAHYNIVTFCCKCD E_744–762_ from the HPV16 E7 protein.^140^ The sugar d-mannose was conjugated (N-terminally) to the LCP molecules to probe the effect of targeting dendritic cell mannose receptors on vaccine efficacy. The vaccines were able to clear or reduce the size of HPV-16 associated tumors in mice, the conjugates bearing mannose causing clearance or reduction in size of tumors to a greater extent than non-mannose-containing vaccines.^140^ LCPs can be prepared by native chemical ligation (NCL), as exemplified in a study in which four copies of the thioester-modified 88/30 GAS peptide antigen (with C to P terminal residue substitution) were ligated using a C-terminal mercaptopropionic acid leucine inker onto an LCP scaffold featuring four cysteine residues.^141^ In another report, multiple peptide epitopes including two 88/30 GAS epitopes, the J8 sequence, and PL1 VLTRRQSQDPKYVTQRIS, an *S. pyogenes* antigen, were attached to an LCP framework using NCL.^142,143^ Studies with mice revealed that high levels of systemic IgG antibodies were elicited against each of the incorporated peptides.^142^ A triepitopic analogue with just one 88/30 GAS peptide attached was also prepared, and its immune response in terms of antibody titers and dendritic cell activation was assayed.^144,145^

## 6 Lipopeptide Micelles

Lipopeptide micelles have been developed as nanoparticles for self-adjuvant vaccines to treat Herpes simplex virus (HSV) infection.^146^ The micelles (presumably spherical) self-assembled from PAs containing HSV envelope glycoprotein B (gB) and glycoprotein D (gD) fragments. The critical micelle concentrations were obtained from fluorescence probe assays, and micelles were sized using dynamic light scattering. The sequences and molecular structures of the PAs are shown in Table 1. The peptide conformation was found from CD spectroscopy to be unordered, both for single and mixed [(C_18_)_2_L-gB_498–505_ and (C_18_)_2_L-gD_301–309_] peptide systems. The micelles (as well as the peptides) were shown to significantly raise *in vitro* levels of cytokines including interleukins (IL-6, IL-8, IL-17, and IL-23), macrophage inflammatory protein (MIP-2), and tissue necrosis factor TNF-α.^146^

**Table 1:**
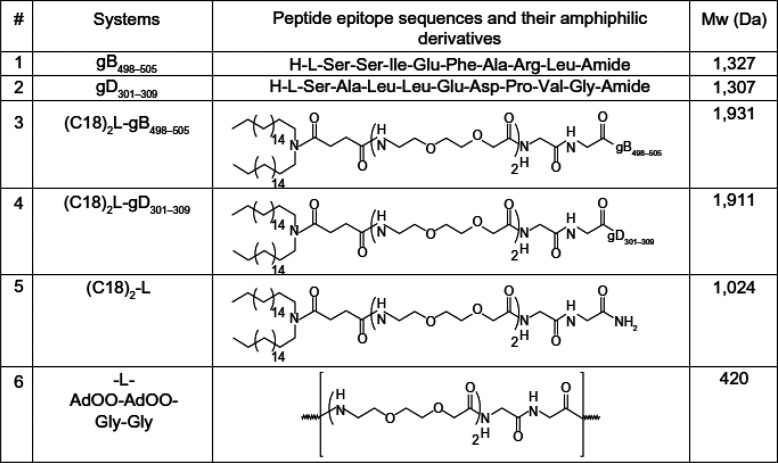
HSV Fragment Peptides gB_498–505_ (**1**) and gD_301–309_ (**2**) and PA Constructs Containing These Fragments (**3**–**5**) That Form HSV-Responsive Immunomodulatory Micelles, as Well as the AdOO Core Structure **6** [AdOO: 8-Amino-3,6-dioxaoctanoic Acid]^a^

a Ref (146) (Copyright Dove Medical Press).

Lipopeptides that form cylindrical micelles have been shown to generate a strong IgG1 antibody response, and these peptide amphiphiles bearing a GAS B cell antigen act as self-adjuvanting vaccines for infections caused by these bacteria.^147^ The peptide having a α-helical structure was conjugated to a dialkyl (dipalmitoyl, di-C_16_) chain. The micelles were shown not to stimulate the pathogen-recognition receptor TLR2 *in vitro*, indicating that this is not the origin of the self-adjuvancy, in contrast to the Pam_3_CSK4 TLR agonist used for comparison (section 4); instead, this was ascribed to the nanoparticle-mediated delivery of the peptide epitope. Thus, the lipopeptide system is non-immunogenic.^147^ The Tirrell group also developed a micelle-forming lipopeptide containing a cytotoxic T cell epitope (SIINFEKL from OVA, discussed in section 4), linked to a di-palmitoyl (diC_16_) chain.^148^ This molecule forms rod-like micelles which have a self-adjuvant property and which were shown to act not via TLR2 but instead they likely enhance uptake by DCs by anchoring of the hydrophobic tails into cell membranes. In addition, the micelles induce an immune response by acting as antigen depots and providing a high density surface presenting the epitopes and reducing degradation.^148^ The lipopeptides induce an antitumor antibody response in mice immunized with the micelles, in particular a reduction in tumor volume, improved survival rate, and cytotoxic T cell response.^148^

## 7 Concluding Remarks

As the studies in this Review make clear, lipopeptides have considerable potential to play a valuable role in the development of vaccines, as antigens and/or adjuvants. There are pros and cons to the use of lipopeptides. Advantages include the ease of design and synthesis (and ability to perform large scale syntheses) of peptides along with the ability to tune conformation and self-assembly by lipidation, using established physicochemical principles. As also highlighted herein, there is scope to design active lipopeptides with a variety of architectures, i.e., configurations of lipids and peptides. In addition, lipopeptides offer the potential to tailor highly specific responses that can be achieved by precision sequence engineering and lipopeptide architecture. Other positives include the ability to prepare peptides at high purity, avoiding biological contamination and reduced allergic response. Disadvantages include the lack of the three-dimensional folded structure of a protein and hence restricted binding properties to human cells compared to actual antigens such as virus coat proteins. In addition, peptides containing native l-amino acid residues are susceptible to proteolysis and they can also have low immunogenicity. The limited stability of peptides *in vivo* can be overcome by a variety of strategies using non-natural amino acids, cyclization, etc.^13,16^ In addition, lipidation enhances stability *in vivo*, since it confers albumin-binding properties which enable longer circulation without degradation.^13,149−153^ Lipopeptides have as yet found limited practical use in vaccines, although a considerable number of peptide and lipopeptide immunotherapies are in development.^16−19^

To be developed for practical uses, immune responses in humans should be studied as part of later stage clinical trials. Most research discussed above has focused on *in vitro* studies and those using rodent models of immune response. However, there can be significant differences in the PRRs presented in human and animal cells. The use of humanized mice or nonhuman primates as better models has thus been proposed. Also, the delivery route for many animal studies differs from the subcutaneous or intramuscular delivery used for practical vaccine delivery.^22^ It has also been pointed out that the dose in many animal studies may differ substantially from that applicable to human use. A further limitation is the use of cell culture methods for *in vitro* studies, since these do not detect inflammatory responses generated by noncirculating tissue cells.^22^

The choice of antigen used together with a particular adjuvant will affect the response of DCs, for example whether a whole protein or fragment antigen such as peptide sequences discussed in this Review is selected. As highlighted in this Review, the presentation of the antigen at the surface of a self-assembled structure such as those formed by lipopeptides can also influence the immune response. In many of the cases discussed in this Review, the self-assembly and conformation of the lipopeptides has not been examined, and this is a subject worth further examination, since self-assembly may have a significant effect on bioactivity.

For successful application, lipopeptide-based adjuvants and antigens will clearly need to have an excellent safety profile. Fortunately, current adjuvanted nonlive vaccines (i.e., the type corresponding to lipopeptides) are considered to generally be insufficiently immunogenic to trigger autoimmunity. This has been well established for the widely used oil-in-water adjuvants MF59 and AS03,^22^ and as discussed herein, some lipopeptide systems have been developed to the stage of clinical trials and do not seem to cause serious adverse reactions.

As mentioned in section 4, TLRs including those that are agonized by lipopeptides such as the Pam_*n*_Cys type are important in COVID-19 pathogenesis,^154,155^ and this has led to a Pam_2_Cys-based intranasal prophylactic INNA-051 which is under commercial development.^128^ This area is likely to be the focus of further intense research activity as the current pandemic continues to have major global effects. As outlined in this Review, lipopeptides also hold great promise in the development of vaccine treatments for many other serious infectious diseases and cancer, and thus have the potential to deliver valuable novel therapeutics based on a diversity of modes of action.

## References

[ref1] Krammer F. (2020) SARS-CoV-2 vaccines in development. Nature 586, 516–527. 10.1038/s41586-020-2798-3. 32967006

[ref2] Ye T. T.; Zhong Z. F.; Garcia-Sastre A.; Schotsaert M.; De Geest B. G. (2020) Current status of COVID-19 (pre)clinical vaccine development. Angew. Chem., Int. Ed. 59, 18885–18897. 10.1002/anie.202008319. PMC7405471 32663348

[ref3] Forni G.; Mantovani A.; Lin C.-C. A. N. (2021) COVID-19 vaccines: where we stand and challenges ahead. Cell Death Differ. 28, 626–639. 10.1038/s41418-020-00720-9. 33479399 PMC7818063

[ref4] Batty C. J.; Heise M. T.; Bachelder E. M.; Ainslie K. M. (2021) Vaccine formulations in clinical development for the prevention of severe acute respiratory syndrome coronavirus 2 infection. Adv. Drug Delivery Rev. 169, 168–189. 10.1016/j.addr.2020.12.006. PMC7733686 33316346

[ref5] Klasse P. J.; Nixon D. F.; Moore J. P. (2021) Immunogenicity of clinically relevant SARS-CoV-2 vaccines in nonhuman primates and humans. Sci. Adv. 7, eabe8065 10.1126/sciadv.abe8065. 33608249 PMC7978427

[ref6] Kashte S.; Gulbake A.; El-Amin S. F.; Gupta A. (2021) COVID-19 vaccines: rapid development, implications, challenges and future prospects. Hum. Cell 34, 711–733. 10.1007/s13577-021-00512-4. 33677814 PMC7937046

[ref7] Löwik D. W. P. M.; van Hest J. C. M. (2004) Peptide based amphiphiles. Chem. Soc. Rev. 33, 234–245. 10.1039/B212638A. 15103405

[ref8] Zhao X. B.; Pan F.; Xu H.; Yaseen M.; Shan H. H.; Hauser C. A. E.; Zhang S. G.; Lu J. R. (2010) Molecular self-assembly and applications of designer peptide amphiphiles. Chem. Soc. Rev. 39, 3480–3498. 10.1039/b915923c. 20498896

[ref9] Matson J. B.; Zha R. H.; Stupp S. I. (2011) Peptide self-assembly for crafting functional biological materials. Curr. Opin. Solid State Mater. Sci. 15, 225–235. 10.1016/j.cossms.2011.08.001. 22125413 PMC3224089

[ref10] Hamley I. W. (2011) Self-Assembly of amphiphilic peptides. Soft Matter 7, 4122–4138. 10.1039/c0sm01218a.

[ref11] Dehsorkhi A.; Castelletto V.; Hamley I. W. (2014) Self-assembling amphiphilic peptides. J. Pept. Sci. 20, 453–467. 10.1002/psc.2633. 24729276 PMC4237179

[ref12] Hamley I. W. (2015) Lipopeptides: from self-assembly to bioactivity. Chem. Commun. 51, 8574–8583. 10.1039/C5CC01535A. 25797909

[ref13] Hamley I. W. Introduction to Peptide Science. Wiley: Chichester, 2020.

[ref14] Durier C.; Launay O.; Meiffredy V.; Saidi Y.; Salmon D.; Levy Y.; Guillet J. G.; Pialoux G.; Aboulker J. P. (2006) Clinical safety of HIV lipopeptides used as vaccines in healthy volunteers and HIV-infected adults. AIDS 20 (7), 1039–1049. 10.1097/01.aids.0000222077.68243.22. 16603857

[ref15] Schmidt J.; Welsch T.; Jager D.; Muhlradt P. F.; Buchler M. W.; Marten A. (2007) Intratumoural injection of the Toll-like receptor-2/6 agonist ’macrophage-activating lipopeptide-2′ in patients with pancreatic carcinoma: a phase I/II trial. Br. J. Cancer 97, 598–604. 10.1038/sj.bjc.6603903. 17667928 PMC2360370

[ref16] Purcell A. W.; McCluskey J.; Rossjohn J. (2007) More than one reason to rethink the use of peptides in vaccine design. Nat. Rev. Drug Discovery 6, 404–414. 10.1038/nrd2224. 17473845

[ref17] Moisa A. A.; Kolesanova E. F. (2010) Synthetic peptide vaccines. Biochem. (Moscow) Suppl. Ser. B: Biomed. Chem. 2010 4, 321–332. 10.1134/S1990750810040025.

[ref18] Skwarczynski M.; Toth I. (2016) Peptide-based synthetic vaccines. Chem. Sci. 7, 842–854. 10.1039/C5SC03892H. 28791117 PMC5529997

[ref19] Malonis R. J.; Lai J. R.; Vergnolle O. (2020) Peptide-based vaccines: Current progress and future challenges. Chem. Rev. 120, 3210–3229. 10.1021/acs.chemrev.9b00472. 31804810 PMC7094793

[ref20] Lu B. L.; Williams G. M.; Verdon D. J.; Dunbar P. R.; Brimble M. A. (2020) Synthesis and evaluation of novel TLR2 agonists as potential adjuvants for cancer vaccines. J. Med. Chem. 63, 2282–2291. 10.1021/acs.jmedchem.9b01044. 31418565

[ref22] Coffman R. L.; Sher A.; Seder R. A. (2010) Vaccine adjuvants: Putting innate immunity to work. Immunity 33, 492–503. 10.1016/j.immuni.2010.10.002. 21029960 PMC3420356

[ref23] Tom J. K.; Albin T. J.; Manna S.; Moser B. A.; Steinhardt R. C.; Esser-Kahn A. P. (2019) Applications of Immunomodulatory Immune Synergies to Adjuvant Discovery and Vaccine Development. Trends Biotechnol. 37 (4), 373–388. 10.1016/j.tibtech.2018.10.004. 30470547

[ref24] Perciani C. T.; Liu L. Y.; Wood L.; MacParland S. A. (2021) Enhancing immunity with nanomedicine: Employing nanoparticles to harness the immune system. ACS Nano 15, 7–20. 10.1021/acsnano.0c08913. 33346646

[ref25] Akira S.; Takeda K. (2004) Toll-like receptor signalling. Nat. Rev. Immunol. 4, 499–511. 10.1038/nri1391. 15229469

[ref26] Takeda K.; Akira S. (2004) Microbial recognition by Toll-like receptors. J. Dermatol. Sci. 34, 73–82. 10.1016/j.jdermsci.2003.10.002. 15033189

[ref27] Broz P.; Monack D. M. (2013) Newly described pattern recognition receptors team up against intracellular pathogens. Nat. Rev. Immunol. 13, 551–565. 10.1038/nri3479. 23846113

[ref28] Moyle P. M.; Toth I. (2013) Modern subunit vaccines: development, components, and research opportunities. ChemMedChem 8, 360–376. 10.1002/cmdc.201200487. 23316023

[ref29] Amanna I. J.; Slifka M. K. (2011) Contributions of humoral and cellular immunity to vaccine-induced protection in humans. Virology 411, 206–215. 10.1016/j.virol.2010.12.016. 21216425 PMC3238379

[ref30] Li X.; Wang X. P.; Ito A. (2018) Tailoring inorganic nanoadjuvants towards next-generation vaccines. Chem. Soc. Rev. 47, 4954–4980. 10.1039/C8CS00028J. 29911725

[ref31] Cox J. C.; Coulter A. R. (1997) Adjuvants - A classification and review of their modes of action. Vaccine 15, 248–256. 10.1016/S0264-410X(96)00183-1. 9139482

[ref32] Singh M.; O’Hagan D. (1999) Advances in vaccine adjuvants. Nat. Biotechnol. 17, 1075–1081. 10.1038/15058. 10545912

[ref33] Gupta R. K.; Relyveld E. H.; Lindblad E. B.; Bizzini B.; Benefraim S.; Gupta C. K. (1993) Adjuvants - a balance between toxicity and adjuvanticity. Vaccine 11, 293–306. 10.1016/0264-410X(93)90190-9. 8447157

[ref34] Aguilar J. C.; Rodriguez E. G. (2007) Vaccine adjuvants revisited. Vaccine 25, 3752–3762. 10.1016/j.vaccine.2007.01.111. 17336431

[ref35] Hu H.-G.; Li Y.-M. (2020) Emerging adjuvants for cancer immunotherapy. Front. Chem. 8, 601 10.3389/fchem.2020.00601. 32850636 PMC7406886

[ref36] Zhang X.; Chentoufi A. A.; Dasgupta G.; Nesburn A. B.; Wu M.; Zhu X.; Carpenter D.; Wechsler S. L.; You S.; BenMohamed L. (2009) A genital tract peptide epitope vaccine targeting TLR-2 efficiently induces local and systemic CD8^+^ T cells and protects against herpes simplex virus type 2 challenge. Mucosal Immunol. 2 (2), 129–143. 10.1038/mi.2008.81. 19129756 PMC4509510

[ref37] Gahéry-Ségard H.; Pialoux G.; Figueiredo S.; Igéa C.; Surenaud M.; Gaston J.; Gras-Masse H.; Lévy J. P.; Guillet J. G. (2003) Long-term specific immune responses induced in humans by a human immunodeficiency virus type 1 lipopeptide-vaccine: Characterization of CD8^+^-T-cell epitopes recognized. J. Virol. 77 (20), 11220–11231. 10.1128/JVI.77.20.11220-11231.2003. 14512570 PMC224965

[ref38] Klinguer C.; David D.; Kouach M.; Wieruszeski J. M.; Tartar A.; Marzin D.; Levy J. P.; Gras-Masse H. (1999) Characterization of a multi-lipopeptides mixture used as an HIV-1 vaccine candidate. Vaccine 18 (3–4), 259–267. 10.1016/S0264-410X(99)00196-6. 10506650

[ref39] Gahéry-Ségard H.; Pialoux G.; Charmeteau B.; Sermet S.; Poncelet H.; Raux M.; Tartar A.; Lévy J. P.; Gras-Masse H.; Guillet J. G. (2000) Multiepitopic B- and T-cell responses induced in humans by a human immunodeficiency virus type 1 lipopeptide vaccine. J. Virol. 74, 1694–1703. 10.1128/JVI.74.4.1694-1703.2000. 10644339 PMC111644

[ref40] Levy Y.; Durier C.; Lascaux A. S.; Meiffrédy V.; Gahéry-Ségard H.; Goujard C.; Rouzioux C.; Resch M.; Guillet J. G.; Kazatchkine M.; Delfraissy J. F.; Aboulker J. P.; Grp A. S. (2006) Sustained control of viremia following therapeutic immunization in chronically HIV-1-infected individuals. AIDS 20, 405–413. 10.1097/01.aids.0000206504.09159.d3. 16439874

[ref41] BenMohamed L.; Thomas A.; Druilhe P. (2004) Long-term multiepitopic cytotoxic-T-lymphocyte responses induced in chimpanzees by combinations of *Plasmodium falciparum* liver-stage peptides and lipopeptides. Infect. Immun. 72, 4376–4384. 10.1128/IAI.72.8.4376-4384.2004. 15271893 PMC470687

[ref42] Zhu X. M.; Ramos T. V.; Gras-Masse H.; Kaplan B. E.; BenMohamed L. (2004) Lipopeptide epitopes extended by an N^ε^-palmitoyl-lysine moiety increase uptake and maturation of dendritic cells through a Toll-like receptor-2 pathway and trigger a Th1-dependent protective immunity. Eur. J. Immunol. 34, 3102–3114. 10.1002/eji.200425166. 15368273

[ref43] Bourel-Bonnet L.; Bonnet D.; Malingue F.; Gras-Masse H.; Melnyk O. (2003) Simultaneous lipidation of a characterized peptide mixture by chemoselective Ligation. Bioconjugate Chem. 14, 494–499. 10.1021/bc0256143. 12643762

[ref44] Bonnet D.; Ollivier N.; Gras-Masse H.; Melnyk O. (2001) Chemoselective acylation of fully deprotected hydrazino acetyl peptides. Application to the synthesis of lipopeptides. J. Org. Chem. 66, 443–449. 10.1021/jo0010577. 11429812

[ref45] Gras-Masse H. (2001) Single-chain lipopeptide vaccines for the induction of virus-specific cytotoxic T cell responses in randomly selected populations. Mol. Immunol. 38 (6), 423–431. 10.1016/S0161-5890(01)00078-5. 11741692

[ref46] Gras-Masse H. (2001) Chemoselective ligation and antigen vectorization. Biologicals 29 (3–4), 183–188. 10.1006/biol.2001.0304. 11851314

[ref47] Zhang X. L.; Issagholian A.; Berg E. A.; Fishman J. B.; Nesburn A. B.; Benmohamed L. (2005) Th-cytotoxic T-lymphocyte chimeric epitopes extended by N-epsilon-palmitoyl lysines induce herpes simplex virus type 1-specific effector CD8(+) Tc-1 responses and protect against ocular infection. J. Virol. 79 (24), 15289–15301. 10.1128/JVI.79.24.15289-15301.2005. 16306600 PMC1316035

[ref48] Moyle P. M.; Toth I. (2008) Self-adjuvanting lipopeptide vaccines. Curr. Med. Chem. 15, 506–516. 10.2174/092986708783503249. 18289006

[ref49] Takeuchi O.; Sato S.; Horiuchi T.; Hoshino K.; Takeda K.; Dong Z. Y.; Modlin R. L.; Akira S. (2002) Cutting edge: Role of Toll-like receptor 1 in mediating immune response to microbial lipoproteins. J. Immunol. 169, 10–14. 10.4049/jimmunol.169.1.10. 12077222

[ref50] Lee H. K.; Lee J.; Tobias P. S. (2002) Two lipoproteins extracted from *Escherichia coli* K-12 LCD25 lipopolysaccharide are the major components responsible for Toll-like receptor 2-mediated signaling. J. Immunol. 168, 4012–4017. 10.4049/jimmunol.168.8.4012. 11937558

[ref51] Hirschfeld M.; Kirschning C. J.; Schwandner R.; Wesche H.; Weis J. H.; Wooten R. M.; Weis J. J. (1999) Cutting edge: Inflammatory signaling by Borrelia burgdorferi lipoproteins is mediated by Toll-like receptor 2. J. Immunol. 163, 2382–2386. 10452971

[ref52] Fisette P. L.; Ram S.; Andersen J. M.; Guo W.; Ingalls R. R. (2003) The Lip lipoprotein from *Neisseria gonorrhoeae* stimulates cytokine release and NF-sB activation in epithelial cells in a Toll-like receptor 2-dependent manner. J. Biol. Chem. 278 (47), 46252–46260. 10.1074/jbc.M306587200. 12966099

[ref53] Luo Y.; Friese O. V.; Runnels H. A.; Khandke L.; Zlotnick G.; Aulabaugh A.; Gore T.; Vidunas E.; Raso S. W.; Novikova E.; Byrne E.; Schlittler M.; Stano D.; Dufield R. L.; Kumar S.; Anderson A. S.; Jansen K. U.; Rouse J. C. (2016) the dual role of lipids of the lipoproteins in Trumenba, a self-adjuvanting vaccine against meningococcal meningitis B disease. AAPS J. 18, 1562–1575. 10.1208/s12248-016-9979-x. 27604766

[ref54] Hashimoto M.; Asai Y.; Ogawa T. (2004) Separation and structural analysis of lipoprotein in a lipopolysaccharide preparation from *Porphyromonas gingivalis*. Int. Immunol. 16, 1431–1437. 10.1093/intimm/dxh146. 15326096

[ref55] Tawaratsumida K.; Furuyashiki M.; Katsumoto M.; Fujimoto Y.; Fukase K.; Suda Y.; Hashimoto M. (2009) Characterization of N-terminal structure of TLR2-activating lipoprotein in *Staphylococcus aureus*. J. Biol. Chem. 284, 9147–9152. 10.1074/jbc.M900429200. 19218237 PMC2666565

[ref56] Nguyen M. T.; Götz F. (2016) Lipoproteins of gram-positive bacteria: key players in the immune response and virulence. Microbiol. Mol. Biol. Rev. 80, 891–903. 10.1128/MMBR.00028-16. 27512100 PMC4981669

[ref57] Zom G. G. P., Khan S., Filippov D. V., and Ossendorp F. TLR ligand-peptide conjugate vaccines: Toward clinical application. In Advances in Immunology, Vol 114: Synthetic Vaccines; Melief C. J. M., Ed.; Elsevier Academic Press Inc: San Diego, CA, 2012; Vol. 114, pp 177–201. 10.1016/B978-0-12-396548-6.00007-X 22449782

[ref58] Lu B. L.; Williams G. M.; Brimble M. A. (2020) TLR2 agonists and their structure-activity relationships. Org. Biomol. Chem. 18, 5073–5094. 10.1039/D0OB00942C. 32582902

[ref59] Zeng W. G.; Ghosh S.; Lau Y. F.; Brown L. E.; Jackson D. C. (2002) Highly immunogenic and totally synthetic lipopeptides as self-adjuvanting immunocontraceptive vaccines. J. Immunol. 169 (9), 4905–4912. 10.4049/jimmunol.169.9.4905. 12391202

[ref60] Hood J. D.; Warshakoon H. J.; Kimbrell M. R.; Shukla N. M.; Malladi S. S.; Wang X. K.; David S. A. (2010) Immunoprofiling toll-like receptor ligands. Comparison of immunostimulatory and proinflammatory profiles in ex vivo human blood models. Hum. Vaccines 6, 322–335. 10.4161/hv.6.4.10866. 20372068

[ref61] Redecke V.; Hacker H.; Datta S. K.; Fermin A.; Pitha P. M.; Broide D. H.; Raz E. (2004) Cutting edge: Activation of Toll-like receptor 2 induces a Th2 immune response and promotes experimental asthma. J. Immunol. 172, 2739–2743. 10.4049/jimmunol.172.5.2739. 14978071

[ref62] Wenink M. H.; Santegoets K. C. M.; Broen J. C. A.; van Bon L.; Abdollahi-Roodsaz S.; Popa C.; Huijbens R.; Remijn T.; Lubberts E.; van Riel P.; van den Berg W. B.; Radstake T. (2009) TLR2 Promotes Th2/Th17 responses via TLR4 and TLR7/8 by abrogating the type I IFN amplification loop. J. Immunol. 183, 6960–6970. 10.4049/jimmunol.0900713. 19915052

[ref63] Aliahmadi E.; Gramlich R.; Grützkau A.; Hitzler M.; Krüger M.; Baumgrass R.; Schreiner M.; Wittig B.; Wanner R.; Peiser M. (2009) TLR2-activated human Langerhans cells promote Th17 polarization via IL-1 beta, TGF-beta and IL-23. Eur. J. Immunol. 39, 1221–1230. 10.1002/eji.200838742. 19350551

[ref64] Reynolds J. M.; Pappu B. P.; Peng J.; Martinez G. J.; Zhang Y. L.; Chung Y.; Ma L.; Yang X. X. O.; Nurieva R. I.; Tian Q.; Dong C. (2010) Toll-like receptor 2 signaling in CD4^+^ T lymphocytes promotes T helper 17 responses and regulates the pathogenesis of autoimmune disease. Immunity 32, 692–702. 10.1016/j.immuni.2010.04.010. 20434372 PMC2878917

[ref65] Imanishi T.; Hara H.; Suzuki S.; Suzuki N.; Akira S.; Saito T. (2007) Cutting edge: TLR2 directly triggers Th1 effector functions. J. Immunol. 178, 6715–6719. 10.4049/jimmunol.178.11.6715. 17513716

[ref66] Jin M. S.; Kim S. E.; Heo J. Y.; Lee M. E.; Kim H. M.; Paik S. G.; Lee H. Y.; Lee J. O. (2007) Crystal structure of the TLR1-TLR2 heterodimer induced by binding of a tri-acylated lipopeptide. Cell 130, 1071–1082. 10.1016/j.cell.2007.09.008. 17889651

[ref67] Kang J. Y.; Nan X.; Jin M. S.; Youn S. J.; Ryu Y. H.; Mah S.; Han S. H.; Lee H.; Paik S. G.; Lee J. O. (2009) Recognition of lipopeptide patterns by Toll-like receptor 2-Toll-like receptor 6 heterodimer. Immunity 31, 873–884. 10.1016/j.immuni.2009.09.018. 19931471

[ref68] Braun V. (1975) Covalent lipoprotein from outer membrane of Escherichia coli. Biochim. Biophys. Acta, Rev. Biomembr. 415, 335–377. 10.1016/0304-4157(75)90013-1. 52377

[ref69] Hantke K.; Braun V. (1973) Covalent binding of lipid to protein - diglyceride and amide-linked fatty-acid at N-terminal end of murein-lipoprotein of *Escherichia coli* outer membrane. Eur. J. Biochem. 34, 284–296. 10.1111/j.1432-1033.1973.tb02757.x. 4575979

[ref70] Bessler W.; Resch K.; Hancock E.; Hantke K. (1977) Induction of lymphocyte-proliferation and membrane changes by lipopeptide derivatives of lipoprotein from outer membrane of Escherichia coli. Z. Immunitätsforsch. 153 (1), 11–22. 10.1016/S0340-904X(77)80023-7. 325934

[ref71] Bessler W. G.; Johnson R. B.; Wiesmüller K.; Jung G. (1982) B-Lymphocyte mitogenicity in vitro of a synthetic lipopeptide fragment derived from bacterial lipoprotein. Hoppe-Seylers Z. Phys. Chem. 363, 767–770. 10.1515/bchm2.1982.363.2.767. 6751983

[ref72] Bessler W. G.; Cox M.; Lex A.; Suhr B.; Wiesmüller K. H.; Jung G. (1985) Synthetic lipopeptide analogs of bacterial lipoprotein are potent polyclonal activators for murine B lymphocytes. J. Immunol. 135, 1900–1905. 3874908

[ref73] Lex A.; Wiesmüller K. H.; Jung G.; Bessler W. G. (1986) A synthetic analogue of Escherichia coli lipoprotein, tripalmitoyl pentapeptide, constitutes a potent immune adjuvant. J. Immunol. 137, 2676–2681. 3531339

[ref74] Reitermann A.; Metzger J.; Wiesmüller K. H.; Jung G.; Bessler W. G. (1989) Lipopeptide derivatives of bacterial lipoprotein constitute potent immune adjuvants combined with or covalently coupled to antigen or hapten. Biol. Chem. Hoppe-Seyler 370, 343–352. 10.1515/bchm3.1989.370.1.343. 2757794

[ref75] Wiesmüller K. H.; Bessler W. G.; Jung G. (1992) Solid-phase peptide-synthesis of lipopeptide vaccines eliciting epitope-specific B-helper, T-helper and T-killer cell response. Int. J. Pept. Protein Res. 40, 255–260. 10.1111/j.1399-3011.1992.tb00299.x. 1282504

[ref76] Sieling P. A.; Chung W.; Duong B. T.; Godowski P. J.; Modlin R. L. (2003) Toll-like receptor 2 ligands as adjuvants for human Th1 responses. J. Immunol. 170, 194–200. 10.4049/jimmunol.170.1.194. 12496400

[ref77] Barrenschee M.; Lex D.; Uhlig S. (2010) Effects of the TLR2 agonists MALP-2 and Pam_3_Cys in isolated mouse lungs. PLoS One 5, e13889 10.1371/journal.pone.0013889. 21124967 PMC2987752

[ref78] Hamley I. W.; Kirkham S.; Dehsorkhi A.; Castelletto V.; Reza M.; Ruokolainen J. (2014) Toll-like receptor agonist lipopeptides self-assemble into distinct nanostructures. Chem. Commun. 50, 15948–15951. 10.1039/C4CC07511K. 25382300

[ref79] Zhao L.; Tu Y. S.; Fang H. P.; Hamley I. W.; Wang Z. W. (2018) Self-assembled micellar structures of lipopeptides with variable number of attached lipid chains revealed by atomistic molecular dynamics simulations. J. Phys. Chem. B 122, 9605–9615. 10.1021/acs.jpcb.8b07877. 30253107

[ref80] Buwitt-Beckmann U.; Heine H.; Wiesmüller K. H.; Jung G.; Brock R.; Ulmer A. J. (2005) Lipopeptide structure determines TLR2 dependent cell activation level. FEBS J. 272, 6354–6364. 10.1111/j.1742-4658.2005.05029.x. 16336272

[ref81] Buwitt-Beckmann U.; Heine H.; Wiesmüller K. H.; Jung G.; Brock R.; Akira S.; Ulmer A. J. (2006) TLR1- and TLR6-independent recognition of bacterial lipopeptides. J. Biol. Chem. 281, 9049–9057. 10.1074/jbc.M512525200. 16455646

[ref82] Buwitt-Beckmann U.; Heine H.; Wiesmüller K. H.; Jung G.; Brock R.; Akira S.; Ulmer A. J. (2005) Toll-like receptor 6-independent signaling by diacylated lipopeptides. Eur. J. Immunol. 35, 282–289. 10.1002/eji.200424955. 15580661

[ref83] Reutter F.; Jung G.; Baier W.; Treyer B.; Bessler W. G.; Wiesmüller K. H. (2005) Immunostimulants and Toll-like receptor ligands obtained by screening combinatorial lipopeptide collections. J. Pept. Res. 65, 375–383. 10.1111/j.1399-3011.2005.00242.x. 15787968

[ref84] Spohn R.; Buwitt-Beckmann U.; Brock R.; Jung G.; Ulmer A. J.; Wiesmüller K. H. (2004) Synthetic lipopeptide adjuvants and Toll-like receptor 2 - structure-activity relationships. Vaccine 22, 2494–2499. 10.1016/j.vaccine.2003.11.074. 15193414

[ref85] Takeuchi O.; Kaufmann A.; Grote K.; Kawai T.; Hoshino K.; Morr M.; Muhlradt P. F.; Akira S. (2000) Preferentially the R-stereoisomer of the mycoplasmal lipopeptide macrophage-activating lipopeptide-2 activates immune cells through a Toll-like receptor 2-and MyD88-dependent signaling pathway. J. Immunol. 164 (2), 554–557. 10.4049/jimmunol.164.2.554. 10623793

[ref86] Metzger J. W.; Wiesmüller K. H.; Jung G. (1991) Synthesis of N-alpha-Fmoc protected derivatives of S-(2,3-dihydroxypropyl)-cysteine and their application in peptide-synthesis. Int. J. Pept. Protein Res. 38 (6), 545–554. 10.1111/j.1399-3011.1991.tb01538.x. 1819589

[ref87] Metzger J.; Jung G.; Bessler W. G.; Hoffmann P.; Strecker M.; Lieberknecht A.; Schmidt U. (1991) Lipopeptides containing 2-(palmitoylamino)-6,7-bis(palmitoyloxy)heptanoic acid - synthesis, stereospecific stimulation of B-lymphocytes and macrophages, and adjuvanticity in vivo and in vitro. J. Med. Chem. 34, 1969–1974. 10.1021/jm00111a008. 2066969

[ref88] Wu W. Y.; Li R. T.; Malladi S. S.; Warshakoon H. J.; Kimbrell M. R.; Amolins M. W.; Ukani R.; Datta A.; David S. A. (2010) Structure-activity relationships in Toll-like receptor-2 agonistic diacylthioglycerol lipopeptides. J. Med. Chem. 53, 3198–3213. 10.1021/jm901839g. 20302301 PMC2859677

[ref89] Agnihotri G.; Crall B. M.; Lewis T. C.; Day T. P.; Balakrishna R.; Warshakoon H. J.; Malladi S. S.; David S. A. (2011) structure-activity relationships in Toll-like receptor 2-agonists leading to simplified monoacyl lipopeptides. J. Med. Chem. 54, 8148–8160. 10.1021/jm201071e. 22007676 PMC3228886

[ref90] Seyberth T.; Voss S.; Brock R.; Wiesmüller K. H.; Jung G. (2006) Lipolanthionine peptides act as inhibitors of TLR2-mediated IL-8 secretion. Synthesis and structure-activity relationships. J. Med. Chem. 49, 1754–1765. 10.1021/jm050585d. 16509590

[ref91] Schromm A. B.; Howe J.; Ulmer A. J.; Wiesmüller K. H.; Seyberth T.; Jung G.; Rossle M.; Koch M. H. J.; Gutsmann T.; Brandenburg K. (2007) Physicochemical and biological analysis of synthetic bacterial lipopeptides - Validity of the concept of endotoxic conformation. J. Biol. Chem. 282, 11030–11037. 10.1074/jbc.M700287200. 17308304

[ref92] Khan S.; Weterings J. J.; Britten C. M.; de Jong A. R.; Graafland D.; Melief C. J. M.; van der Burg S. H.; van der Marel G.; Overkleeft H. S.; Filippov D. V.; Ossendorp F. (2009) Chirality of TLR-2 ligand Pam_3_CysSK_4_ in fully synthetic peptide conjugates critically influences the induction of specific CD8^+^ T-cells. Mol. Immunol. 46, 1084–1091. 10.1016/j.molimm.2008.10.006. 19027958

[ref93] Willems M.; Zom G. G.; Khan S.; Meeuwenoord N.; Melief C. J. M.; van der Stelt M.; Overkleeft H. S.; Codee J. D. C.; van der Marel G. A.; Ossendorp F.; Filippov D. V. (2014) N-Tetradecylcarbamyl lipopeptides as novel agonists for Toll-like receptor 2. J. Med. Chem. 57, 6873–6878. 10.1021/jm500722p. 25019313

[ref94] Hioe C. E.; Qiu H.; Chend P. D.; Bian Z. N.; Li M. L.; Li J.; Singh M.; Kuebler P.; McGee P.; Ohagan D.; Zamb T.; Koff W.; Allsopp C.; Wang C. Y.; Nixon D. F. (1996) Comparison of adjuvant formulations for cytotoxic T cell induction using synthetic peptides. Vaccine 14, 412–418. 10.1016/0264-410X(95)00191-3. 8735553

[ref95] Kleine B.; Rapp W.; Wiesmüller K. H.; Edinger M.; Beck W.; Metzger J.; Ataulakhanov R.; Jung G.; Bessler W. G. (1994) Lipopeptide-polyoxyethylene conjugates as mitogens and adjuvants. Immunobiology 190, 53–66. 10.1016/S0171-2985(11)80283-4. 8082887

[ref96] https://sapvaxllc.com/.

[ref97] Seth A.; Yasutomi Y.; Jacoby H.; Callery J. C.; Kaminsky S. M.; Koff W. C.; Nixon D. F.; Letvin N. L. (2000) Evaluation of a lipopeptide immunogen as a therapeutic in HIV type 1-seropositive individuals. AIDS Res. Hum. Retroviruses 16, 337–343. 10.1089/088922200309214. 10716371

[ref98] Wright T. H.; Brooks A. E. S.; Didsbury A. J.; Williams G. M.; Harris P. W. R.; Dunbar P. R.; Brimble M. A. (2013) Direct peptide lipidation through thiol-ene coupling enables rapid synthesis and evaluation of self-adjuvanting vaccine candidates. Angew. Chem., Int. Ed. 52, 10616–10619. 10.1002/anie.201305620. 23939951

[ref99] Wright T. H.; Brooks A. E. S.; Didsbury A. J.; MacIntosh J. D.; Williams G. M.; Harris P. W. R.; Dunbar P. R.; Brimble M. A. (2013) Direct peptide lipidation through thiol-ene coupling enables rapid synthesis and evaluation of self-adjuvanting vaccine candidates (erratum). Angew. Chem., Int. Ed. 52, 11686–11686. 10.1002/anie.201308276. 23939951

[ref100] Kopycinski J.; Osman M.; Griffiths P. D.; Emery V. C. (2010) Sequence flexibility of the immunodominant HLA A*0201 restricted ppUL83 CD8 T-Cell epitope of human cytomegalovirus. J. Med. Virol. 82, 94–103. 10.1002/jmv.21668. 19950243

[ref101] Yang S. H.; Harris P. W. R.; Williams G. M.; Brimble M. A. (2016) Lipidation of cysteine or cysteine-containing peptides using the thiol-ene reaction (CLipPA). Eur. J. Org. Chem. 2016, 2608–2616. 10.1002/ejoc.201501375.

[ref102] Chua B. Y.; Pejoski D.; Turner S. J.; Zeng W. G.; Jackson D. C. (2011) Soluble Proteins Induce Strong CD8^+^ T cell and antibody responses through electrostatic association with simple cationic or anionic lipopeptides that target TLR2. J. Immunol. 187, 1692–1701. 10.4049/jimmunol.1100486. 21742967

[ref103] Wijayadikusumah A. R.; Sullivan L. C.; Jackson D. C.; Chua B. Y. (2017) Structure-function relationships of protein-lipopeptide complexes and influence on immunogenicity. Amino Acids 49, 1691–1704. 10.1007/s00726-017-2466-6. 28718065

[ref104] Khan S.; Bijker M. S.; Weterings J. J.; Tanke H. J.; Adema G. J.; van Hall T.; Drijfhout J. W.; Melief C. J. M.; Overkleeft H. S.; van der Marel G. A.; Filippov D. V.; van der Burg S. H.; Ossendorp F. (2007) Distinct uptake mechanisms but similar intracellular processing of two different Toll-like receptor ligand-peptide conjugates in dendritic cells. J. Biol. Chem. 282, 21145–21159. 10.1074/jbc.M701705200. 17462991

[ref105] Lau Y. F.; Deliyannis G.; Zeng W. G.; Mansell A.; Jackson D. C.; Brown L. E. (2006) Lipid-containing mimetics of natural triggers of innate immunity as CTL-inducing influenza vaccines. Int. Immunol. 18, 1801–1813. 10.1093/intimm/dxl114. 17077175

[ref106] Zeng W. G.; Azzopardi K.; Hocking D.; Wong C. Y.; Robevska G.; Tauschek M.; Robins-Browne R. M.; Jackson D. C. (2012) A totally synthetic lipopeptide-based self-adjuvanting vaccine induces neutralizing antibodies against heat-stable enterotoxin from enterotoxigenic Escherichia coli. Vaccine 30, 4800–4806. 10.1016/j.vaccine.2012.05.017. 22634295

[ref107] Taxt A.; Aasland R.; Sommerfelt H.; Nataro J.; Puntervoll P. (2010) Heat-Stable enterotoxin of Enterotoxigenic Escherichia coli as a vaccine target. Infect. Immun. 78, 1824–1831. 10.1128/IAI.01397-09. 20231404 PMC2863518

[ref108] Zeng W. G.; Horrocks K. J.; Robevska G.; Wong C. Y.; Azzopardi K.; Tauschek M.; Robins-Browne R. M.; Jackson D. C. (2011) A modular approach to assembly of totally synthetic self-adjuvanting lipopeptide-based vaccines allows conformational epitope building. J. Biol. Chem. 286, 12944–12951. 10.1074/jbc.M111.227744. 21321114 PMC3075641

[ref109] Deliyannis G.; Kedzierska K.; Lau Y. F.; Zeng W. G.; Turner S. J.; Jackson D. C.; Brown L. E. (2006) Intranasal lipopeptide primes lung-resident memory CD8^+^ T cells for long-term pulmonary protection against influenza. Eur. J. Immunol. 36, 770–778. 10.1002/eji.200535217. 16435281

[ref110] Zeng W. G.; Eriksson E. M.; Lew A.; Jackson D. C. (2011) Lipidation of intact proteins produces highly immunogenic vaccine candidates. Mol. Immunol. 48, 490–496. 10.1016/j.molimm.2010.10.003. 21056473

[ref111] Jackson D. C.; Lau Y. F.; Le T.; Suhrbier A.; Deliyannis G.; Cheers C.; Smith C.; Zeng W. G.; Brown L. E. (2004) A totally synthetic vaccine of generic structure that targets Toll-like receptor 2 on dendritic cells and promotes antibody or cytotoxic T cell responses. Proc. Natl. Acad. Sci. U. S. A. 101, 15440–15445. 10.1073/pnas.0406740101. 15489266 PMC523460

[ref112] Chua B. Y.; Zeng W.; Jackson D. C. (2008) Peptide-based drug design. Methods Mol. Biol. 494, 247–261. 10.1007/978-1-59745-419-3_14. 18726578

[ref113] Schultz T. E.; Wiesmüller K. H.; Lucas M.; Dobos K. M.; Baxter A. G.; Blumenthal A. (2018) The N-terminal peptide moiety of the Mycobacterium tuberculosis 19 kDa lipoprotein harbors RP105-agonistic properties. J. Leukocyte Biol. 103, 311–319. 10.1002/JLB.2MA0517-190RR. 29345364

[ref114] Radolf J. D.; Norgard M. V.; Brandt M. E.; Isaacs R. D.; Thompson P. A.; Beutler B. (1991) Lipoproteins of Borrelia burgdorferi and Treponema pallidum activate cachectin tumor-necrosis-factor synthesis - analysis using a cat reporter construct. J. Immunol. 147, 1968–1974. 1890308

[ref115] Sellati T. J.; Bouis D. A.; Kitchens R. L.; Darveau R. P.; Pugin J.; Ulevitch R. J.; Gangloff S. C.; Goyert S. M.; Norgard M. V.; Radolf J. D. (1998) Treponema pallidum and Borrelia burgdorferi lipoproteins and synthetic lipopeptides activate monocytic cells via a CD14-dependent pathway distinct from that used by lipopolysaccharide. J. Immunol. 160, 5455–5464. 9605148

[ref116] Erdile L. F.; Guy B. (1997) OspA lipoprotein of Borrelia burgdorferi is a mucosal immunogen and adjuvant. Vaccine 15, 988–995. 10.1016/S0264-410X(96)00295-2. 9261945

[ref117] Fonseca D.; Joosten D.; Snippe H.; Verheul A. F. M. (2000) Evaluation of T-cell responses to peptides and lipopeptides with MHC class I binding motifs derived from the amino acid sequence of the19-kDa lipoprotein of *Mycobacterium tuberculosis*. Mol. Immunol. 37, 413–422. 10.1016/S0161-5890(00)00066-3. 11090876

[ref118] Aiga T.; Manabe Y.; Ito K.; Chang T. C.; Kabayama K.; Ohshima S.; Kametani Y.; Miura A.; Furukawa H.; Inaba H.; Matsuura K.; Fukase K. (2020) Immunological evaluation of co-assembling a lipidated peptide antigen and lipophilic adjuvants: self-adjuvanting anti-breast-cancer vaccine candidates. Angew. Chem., Int. Ed. 59, 17705–17711. 10.1002/anie.202007999. 32583549

[ref119] Castelletto V.; Kirkham S.; Hamley I. W.; Kowalczyk R.; Rabe M.; Reza M.; Ruokolainen J. (2016) Self-assembly of the Toll-like receptor agonist macrophage-activating lipopeptide MALP-2 and of its constituent peptide. Biomacromolecules 17, 631–640. 10.1021/acs.biomac.5b01573. 26752598

[ref120] Mühlradt P. F.; Kiess M.; Meyer H.; Süssmuth R.; Jung G. (1997) Isolation, structure elucidation, and synthesis of a macrophage stimulatory lipopeptide from *Mycoplasma fermentans* acting at picomolar concentration. J. Exp. Med. 185, 1951–1958. 10.1084/jem.185.11.1951. 9166424 PMC2196331

[ref121] Takeuchi O.; Kawai T.; Muhlradt P. F.; Morr M.; Radolf J. D.; Zychlinsky A.; Takeda K.; Akira S. (2001) Discrimination of bacterial lipoproteins by Toll-like receptor 6. Int. Immunol. 13, 933–940. 10.1093/intimm/13.7.933. 11431423

[ref122] Morr M.; Takechi O.; Akira S.; Simon M. M.; Mühlradt P. F. (2002) Differential recognition of structural details of bacterial lipopeptides by Toll-like receptors. Eur. J. Immunol. 32, 3337–3347. 10.1002/1521-4141(2002012)32:12<3337::AID-IMMU3337>3.0.CO;2-I. 12432564

[ref123] Mühlradt P. F.; Kiess M.; Meyer H.; Süssmuth R.; Jung G. (1998) Structure and specific activity of macrophage-stimulating lipopeptides from *Mycoplasma hyorhinis*. Infect. Immun. 66, 4804–4810. 10.1128/IAI.66.10.4804-4810.1998. 9746582 PMC108593

[ref124] Cataldi A.; Yevsa T.; Vilte D. A.; Schulze K.; Castro-Parodi M.; Larzabal M.; Ibarra C.; Mercado E. C.; Guzman C. A. (2008) Efficient immune responses against Intimin and EspB of enterohaernorragic *Escherichia coli* after intranasal vaccination using the TLR2/6 agonist MALP-2 as adjuvant. Vaccine 26, 5662–5667. 10.1016/j.vaccine.2008.07.027. 18675866

[ref125] Shibata K.; Hasebe A.; Into T.; Yamada M.; Watanabe T. (2000) The N-terminal lipopeptide of a 44-kDa membrane-bound lipoprotein of Mycoplasma salivarium is responsible for the expression of intercellular adhesion molecule-1 on the cell surface of normal human gingival fibroblasts. J. Immunol. 165, 6538–6544. 10.4049/jimmunol.165.11.6538. 11086096

[ref126] Nakamura J.; Shibata K.; Hasebe A.; Into T.; Watanabe T.; Ohata N. (2002) Signaling pathways induced by lipoproteins derived from *Mycoplasma salivarium* and a synthetic lipopeptide (FSL-1) in normal human gingival fibroblasts. Microbiol. Immunol. 46, 151–158. 10.1111/j.1348-0421.2002.tb02680.x. 12008923

[ref127] Okusawa T.; Fujita M.; Nakamura J. I.; Into T.; Yasuda M.; Yoshimura A.; Hara Y.; Hasebe A.; Golenbock D. T.; Morita M.; Kuroki Y.; Ogawa T.; Shibata K. I. (2004) Relationship between structures and biological activities of mycoplasmal diacylated lipopeptides and their recognition by Toll-like receptors 2 and 6. Infect. Immun. 72, 1657–1665. 10.1128/IAI.72.3.1657-1665.2004. 14977973 PMC355991

[ref128] Proud P. C. (2021) Prophylactic intranasal administration of a TLR2/6 agonist reduces upper respiratory tract viral shedding in a SARS-CoV-2 challenge ferret model. EBioMedicine 63, 103153 10.1016/j.ebiom.2020.103153. 33279857 PMC7711201

[ref129] Nguyen D. T.; de Witte L.; Ludlow M.; Yuksel S.; Wiesmuller K. H.; Geijtenbeek T. B. H.; Osterhaus A.; de Swart R. L. (2010) The synthetic bacterial lipopeptide Pam3CSK4 modulates respiratory syncytial virus infection independent of tlr activation. PLoS Pathog. 6, e1001049 10.1371/journal.ppat.1001049. 20808895 PMC2924323

[ref130] Zheng M.; Karki R.; Williams E. P.; Yang D.; Fitzpatrick E.; Vogel P.; Jonsson C. B.; Kanneganti T.-D. (2021) TLR2 senses the SARS-CoV-2 envelope protein to produce inflammatory cytokines. Nat. Immunol. 10.1038/s41590-021-00937-x. PMC8882317 33963333

[ref131] Weterings J. J.; Khan S.; van der Heden G. J.; Drijfhout J. W.; Melief C. J. M.; Overkleeft H. S.; van der Burg O. H.; Ossendorp F.; van der Marel G. A.; Filippov D. V. (2006) Synthesis of 2-alkoxy-8-hydroxyadenylpeptides: Towards synthetic epitope-based vaccines. Bioorg. Med. Chem. Lett. 16, 3258–3261. 10.1016/j.bmcl.2006.03.034. 16581248

[ref132] Zhong W.; Skwarczynski M.; Toth I. (2009) Lipid core peptide system for gene, drug, and vaccine delivery. Aust. J. Chem. 62, 956–967. 10.1071/CH09149.

[ref133] Skwarczynski M.; Dougall A. M.; Khoshnejad M.; Chandrudu S.; Pearson M. S.; Loukas A.; Toth I. (2012) Peptide-based subunit vaccine against hookworm infection. PLoS One 7, e46870 10.1371/journal.pone.0046870. 23056500 PMC3463534

[ref134] Abdel-Aal A. B. M.; Batzloff M. R.; Fujita Y.; Barozzi N.; Faria A.; Simerska P.; Moyle P. M.; Good M. F.; Toth I. (2008) Structure-activity relationship of a series of synthetic lipopeptide self-adjuvanting group A streptococcal vaccine candidates. J. Med. Chem. 51, 167–172. 10.1021/jm701091d. 18072728

[ref135] Abdel-Aal A. B. M.; Zaman M.; Fujita Y.; Batzloff M. R.; Good M. F.; Toth I. (2010) Design of three-component vaccines against group a streptococcal infections: importance of spatial arrangement of vaccine components. J. Med. Chem. 53, 8041–8046. 10.1021/jm1007787. 21028828

[ref136] Zaman M.; Abdel-Aal A. M.; Phillipps K. S. M.; Fujita Y.; Good M. F.; Toth I. (2010) Structure-activity relationship of lipopeptide Group A streptococcus (GAS) vaccine candidates on Toll-like receptor 2. Vaccine 28, 2243–2248. 10.1016/j.vaccine.2009.12.046. 20045502

[ref137] Zaman M.; Abdel-Aal A. B. M.; Fujita Y.; Phillipps K. S. M.; Batzloff M. R.; Good M. F.; Toth I. (2012) Immunological evaluation of Lipopeptide Group A streptococcus (GAS) vaccine: structure-activity relationship. PLoS One 7, e30146 10.1371/journal.pone.0030146. 22253911 PMC3257266

[ref138] Hayman W. A.; Toth I.; Flinn N.; Scanlon M.; Good M. F. (2002) Enhancing the immunogenicity and modulating the fine epitope recognition of antisera to a helical group A streptococcal peptide vaccine candidate from the M protein using lipid-core peptide technology. Immunol. Cell Biol. 80, 178–187. 10.1046/j.1440-1711.2002.01067.x. 11940119

[ref139] Olive C.; Sun H. K.; Ho M. F.; Dyer J.; Horvath A.; Toth I.; Good M. F. (2006) Intranasal administration is an effective mucosal vaccine delivery route for self-adjuvanting lipid core peptides targeting the group A streptococcal M protein. J. Infect. Dis. 194, 316–324. 10.1086/505580. 16826479

[ref140] Moyle P. M.; Olive C.; Ho M. F.; Pandey M.; Dyer J.; Suhrbier A.; Fujita Y.; Toth I. (2007) Toward the development of prophylactic and therapeutic human papillomavirus type-16 lipopeptide vaccines. J. Med. Chem. 50, 4721–4727. 10.1021/jm070287b. 17705361

[ref141] Moyle P. M.; Hari Y.; Huang N.; Olive C.; Good M. F.; Toth I. (2007) A technique for the synthesis of highly-pure, mono-epitopic, multi-valent lipid core peptide vaccines. Tetrahedron Lett. 48, 4965–4967. 10.1016/j.tetlet.2007.05.129.

[ref142] Moyle P. M.; Olive C.; Good M. F.; Toth I. (2006) Method for the synthesis of highly pure vaccines using the lipid core peptide system. J. Pept. Sci. 12 (12), 800–807. 10.1002/psc.815. 17131293

[ref143] Moyle P. M.; Olive C.; Ho M. F.; Burgess M.; Karpati L.; Good M. F.; Toth I. (2006) Method for the synthesis of multi-epitopic Streptococcus pyogenes lipopeptide vaccines using native chemical ligation. J. Org. Chem. 71, 6846–6850. 10.1021/jo060960p. 16930036

[ref144] Moyle P. M.; Olive C.; Ho M. F.; Good M. F.; Toth I. (2006) Synthesis of a highly pure lipid core peptide based self-adjuvanting triepitopic group A Streptococcal vaccine, and subsequent immunological evaluation. J. Med. Chem. 49, 6364–6370. 10.1021/jm060475m. 17034142

[ref145] Phillipps K. S. M.; Wykes M. N.; Liu X. Q.; Brown M.; Blanchfield J.; Toth I. (2009) A novel synthetic adjuvant enhances dendritic cell function. Immunology 128, e582–e588. 10.1111/j.1365-2567.2008.03038.x. 19740319 PMC2753928

[ref146] Accardo A.; Vitiello M.; Tesauro D.; Galdiero M.; Finamore E.; Martora F.; Mansi R.; Ringhieri P.; Morelli G. (2014) Self-assembled or mixed peptide amphiphile micelles from Herpes simplex virus glycoproteins as potential immunomodulatory treatment. Int. J. Nanomed. 9, 2137–2148. 10.2147/IJN.S57656. PMC4019629 24855352

[ref147] Trent A.; Ulery B. D.; Black M. J.; Barrett J. C.; Liang S.; Kostenko Y.; David N. A.; Tirrell M. V. (2015) Peptide amphiphile micelles self-adjuvant group a streptococcal vaccination. AAPS J. 17, 380–388. 10.1208/s12248-014-9707-3. 25527256 PMC4365084

[ref148] Black M.; Trent A.; Kostenko Y.; Lee J. S.; Olive C.; Tirrell M. (2012) Self-assembled peptide amphiphile micelles containing a cytotoxic T-Cell epitope promote a protective immune response in vivo. Adv. Mater. 24 (28), 3845–3849. 10.1002/adma.201200209. 22550019

[ref149] Knudsen L. B.; Nielsen P. F.; Huusfeldt P. O.; Johansen N. L.; Madsen K.; Pedersen F. Z.; Thogersen H.; Wilken M.; Agerso H. (2000) Potent derivatives of glucagon-like peptide-1 with pharmacokinetic properties suitable for once daily administration. J. Med. Chem. 43, 1664–1669. 10.1021/jm9909645. 10794683

[ref150] Drucker D. J.; Dritselis A.; Kirkpatrick P. (2010) Liraglutide. Nat. Rev. Drug Discovery 9, 267–268. 10.1038/nrd3148. 20357801

[ref151] Bellmann-Sickert K.; Elling C. E.; Madsen A. N.; Little P. B.; Lundgren K.; Gerlach L. O.; Bergmann R.; Holst B.; Schwartz T. W.; Beck-Sickinger A. G. (2011) Long-acting lipidated analogue of human pancreatic polypeptide is slowly released into circulation. J. Med. Chem. 54, 2658–2667. 10.1021/jm101357e. 21410292

[ref152] Hutchinson J. A.; Burholt S.; Hamley I. W. (2017) Peptide hormones and lipopeptides: from self-assembly to therapeutic applications. J. Pept. Sci. 23, 82–94. 10.1002/psc.2954. 28127868 PMC5324658

[ref153] Hutchinson J. A.; Burholt S.; Hamley I. W.; Lundback A.-K.; Uddin S.; dos Santos A. G.; Reza M.; Seitsonen J.; Ruokolainen J. (2018) The effect of lipidation on the self-Assembly of the gut-derived peptide hormone PYY_3–36_. Bioconjugate Chem. 29, 2296–2308. 10.1021/acs.bioconjchem.8b00286. 29856926

[ref154] Patra R.; Das N. C.; Mukherjee S. (2021) Targeting human TLRs to combat COVID-19: A solution?. J. Med. Virol. 93, 615–617. 10.1002/jmv.26387. 32749702 PMC7436140

[ref155] Khanmohammadi S.; Rezaei N. (2021) Role of Toll-like receptors in the pathogenesis of COVID-19. J. Med. Virol. 93, 2735–2739. 10.1002/jmv.26826. 33506952 PMC8014260

